# Ringed seal (*Pusa hispida*) seasonal movements, diving, and haul‐out behavior in the Beaufort, Chukchi, and Bering Seas (2011–2017)

**DOI:** 10.1002/ece3.6302

**Published:** 2020-05-05

**Authors:** Andrew L. Von Duyke, David C. Douglas, Jason K. Herreman, Justin A. Crawford

**Affiliations:** ^1^ Department of Wildlife Management North Slope Borough Barrow AK USA; ^2^ U.S. Geological Survey Alaska Science Center Juneau AK USA; ^3^ Alaska Department of Fish and Game Arctic Marine Mammal Program Fairbanks AK USA; ^4^ Present address: Alaska Department of Fish and Game Homer AK USA

**Keywords:** Alaska, Arctic, ecotype, marine mammals, phocid, satellite telemetry, spatial ecology, unusual mortality event

## Abstract

Continued Arctic warming and sea‐ice loss will have important implications for the conservation of ringed seals, a highly ice‐dependent species. A better understanding of their spatial ecology will help characterize emerging ecological trends and inform management decisions. We deployed satellite transmitters on ringed seals in the summers of 2011, 2014, and 2016 near Utqiaġvik (formerly Barrow), Alaska, to monitor their movements, diving, and haul‐out behavior. We present analyses of tracking and dive data provided by 17 seals that were tracked until at least January of the following year. Seals mostly ranged north of Utqiaġvik in the Beaufort and Chukchi Seas during summer before moving into the southern Chukchi and Bering Seas during winter. In all seasons, ringed seals occupied a diversity of habitats and spatial distributions, from near shore and localized, to far offshore and wide‐ranging in drifting sea ice. Continental shelf waters were occupied for >96% of tracking days, during which repetitive diving (suggestive of foraging) primarily to the seafloor was the most frequent activity. From mid‐summer to early fall, 12 seals made ~1‐week forays off‐shelf to the deep Arctic Basin, most reaching the retreating pack‐ice, where they spent most of their time hauled out. Diel activity patterns suggested greater allocation of foraging efforts to midday hours. Haul‐out patterns were complementary, occurring mostly at night until April‐May when midday hours were preferred. Ringed seals captured in 2011—concurrent with an unusual mortality event that affected all ice‐seal species—differed morphologically and behaviorally from seals captured in other years. Speculations about the physiology of molting and its role in energetics, habitat use, and behavior are discussed; along with possible evidence of purported ringed seal ecotypes.

## Graphical abstract

Arctic warming and sea‐ice loss will have implications for the conservation of ice‐dependent ringed seals. A better understanding of their spatial ecology will help characterize emerging ecological trends and benefit management decisions. We present analyses of ringed seal tracking and dive data (*n* = 17). Activities occurring on varying temporal scales are described, as are seasonal movements, and habitat use. Finally, speculation on molting physiology and its role in energetics, habitat use, and behavior are discussed, along with speculation about two purported ringed seal ecotypes.

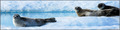

## 1 INTRODUCTION

Arctic species face significant ecological challenges owing to rapid climate change. Arctic warming is occurring at twice the global rate (Arctic Monitoring & Assessment Programme, 2017; Overland et al., 2016), and sea‐ice loss is outpacing model predictions (Kwok, 2018; Maslanik, Stroeve, Fowler, & Emery, 2011; Stroeve, Holland, Meier, Scambos, & Serrez, 2007; Stroeve & Notz, 2018; Timmermans, Toole, & Krishfield, 2018). Changing sea‐ice dynamics are expected to have substantial ecological implications (Arrigo, van Dijken, & Pabi, 2008; Grebmeier et al., 2006; Hoegh‐Guldberg & Bruno, 2010) exacerbated by increased disturbances associated with expanding industrial development and commercial shipping (Harsem, Heen, Rodrigues, & Vassdal, 2015; Huntington, 2009; Smith & Stephenson, 2013). Spatiotemporal variability in the rate and magnitude of change in the Arctic adds additional complexity (Kovacs, Lydersen, Overland, & Moore, 2011). Although Arctic species can serve as sentinels of change (Moore, 2008), knowledge gaps persist in the understanding of many species’ basic biology, and addressing those gaps will improve efforts to identify, understand, manage, and adapt to the effects of rapidly changing environmental conditions.

**FIGURE 1:**
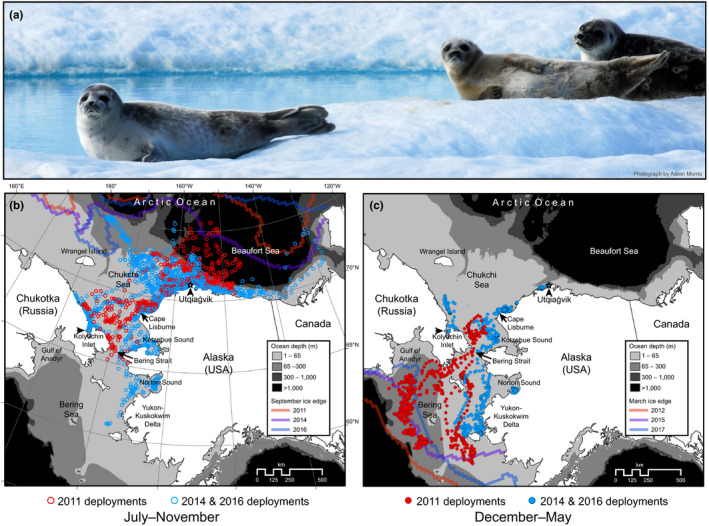
(a) Three ringed seals (*Pusa hispida*) hauled out on multi‐year ice in the southern Beaufort Sea near Utqiaġvik, AK. Note the molting fur on the center seal and the black face of a “rutting” male on the right. Daily CRAWL location estimates (*n* = 4,083) of the 17 ringed seals instrumented with satellite tracking tags are shown for July–September (b) and December–May (c). Colors distinguish seals tagged in 2011 (red, *n* = 5) from those tagged in 2014 and 2016 (blue, *n* = 2 and 10 respectively). The light gray contour at the 65 m isobath corresponds to the vertical line in Figure 7. The colored lines in (b) and (c) indicate the minimum and maximum extent of the sea ice in September and March, respectively, for each of the three tagging periods

Ringed seals (*Pusa hispida*; Figure 1a) are a small, highly abundant phocid with a circumpolar distribution (Burns, 1970; McLaren, 1958). They are an important component of the Arctic food web as a generalist predator (Crawford, Quakenbush, & Citta, 2015; Dehn et al., 2007; Lowry, Frost, & Burns, 1980), the primary prey of polar bears (*Ursus maritimus*; Stirling & Archibald, 1977), and a valuable subsistence resource for coastal Inuit people. Ringed seals are considered the most ice dependent of the four “ice associated” seal species in the western Arctic (Smith, Stirling, & Taugbøl 1991), which also include: bearded seals (*Erignathus barbatus*), spotted seals (*Phoca largha*), and ribbon seals (*Histriophoca fasciata*). They are well adapted to wintering within shore fast and pack‐ice habitats—using their front claws to maintain breathing holes and to excavate lairs in snow that has drifted above these holes (Stirling, 1977). Female ringed seals give birth to and nurse their pups within snow lairs, which are important to pup survival because they provide shelter from the elements and concealment from predators (Smith, 1976; Stirling & Archibald, 1977; Smith, 1980; Smith, Stirling, & Taugbøl 1991; Stirling & Smith, 2004). Sea ice also serves as a platform on which ringed seals haul out during their annual pelage molt in spring (Fay, 1974; Smith & Stirling, 1975)—a time when their epidermis and fur are shed and replaced. This close relationship with sea ice suggests that ringed seals may be sensitive to changes in their habitat (Laidre et al., 2008). Given the ecological importance of ringed seals and the ongoing rapid changes to their sea‐ice habitats, a characterization of ringed seal movements, diving, and haul‐out behavior has practical applications for their management and can contribute to a better understanding of emerging ecological trends in the Arctic.

Previous studies of ringed seal movements in the Beaufort‐Chukchi‐Bering (BCB) Sea region reported seasonal and demographic movement patterns (Crawford, Frost, Quakenbush, & Whiting, 2012, 2018; Harwood, Smith, & Auld, 2012; Harwood, Smith, Auld, Melling, & Yurkowski, 2015; Kelly et al., 2010), though broad‐scale variability in these patterns appeared to be associated with capture location. For example, ringed seals tagged during September 2001 and 2002 just east of the Beaufort Sea in the southwestern Canadian Archipelago (Harwood et al., 2012) made extensive autumn movements westward that terminated primarily in the western Chukchi Sea north of Chukotka (Russia), whereas seals tagged nearby (~250 km to the east) in June and July of 1999, 2000 and 2010 (Harwood et al., 2015) exhibited more localized movements and remained in the southwestern Archipelago throughout autumn and winter. Though similar in phenology, ringed seals captured in Kotzebue Sound, Alaska (Crawford, Frost, et al., 2012; Crawford et al., 2018) moved into the southern Chukchi and Bering seas during winter. Given the enormity of the BCB, the limited number of tracking studies to date, and the apparent differences in spatial distribution and movements associated with tagging locations, there remains a need to document and characterize the movements and behaviors of ringed seals from other locales within the BCB to more fully document this species’ spatial ecology.

Here, we present seasonal movements, habitat use, diving, and haul‐out behavior of ringed seals instrumented with satellite transmitters in the vicinity of Utqiaġvik (formerly Barrow), Alaska—a capture region not represented in prior tracking studies. We illustrate and quantify their movements and behaviors with respect to several geographic and demographic covariates. This work contributes to a growing body of literature about ringed seal spatial use, while also informing broader scale Arctic ecosystem monitoring efforts (Moore et al., 2014).

## 2 METHODS

Ringed seals were captured near Utqiaġvik, AK (71.3° N, 156.8° W) during June–July of 2011, 2014, and 2016. All captures were made with nets that were set and continuously monitored near ice floes where seals had been observed. All nets had a lightweight lead‐line and a highly visible float‐line to ensure that entangled ringed seals could surface to breathe and that observers could readily determine when a capture occurred.

Upon capture, the seals were physically restrained during sampling and instrumentation. Biometric and demographic data were recorded (Table 1; Appendix A), including body mass, standard length, axillary girth, sex, and age class (Geraci & Lownsbury, 2005). Age was determined by counting the alternating light and dark bands on the front claws. Seals with ≥5 claw bands were classified as adults (McLaren, 1958); otherwise, they were classified as subadults. One seal with no record of claw bands was designated as a subadult based on its small size and weight (Crawford, Frost, et al., 2012).

**TABLE 1:** Attributes and tracking duration for 17 ringed seals marked with satellite transmitters during the summer near Utqiaġvik, AK

Seal ID	Sex	Age class	Weight (kg)	Length (cm)	Ax. Girth (cm)	Claw bands	First loc	Last loc	Elapsed days	CRAWL days
PH2011BW03^a^	M	Subadult	24.8	95	84	4+	16‐July‐2011	09‐June‐2012	330	330
PH2011BW10^a^	F	Adult	26.6	92	72	8+	21‐July‐2011	01‐May‐2012	286	276
PH2011BW11^a^	F	Adult	23.2	93	76	7+	22‐July‐2011	04‐June‐2012	319	307
PH2011BW12^a^	M	Adult	27.2	92	81	8+	22‐July‐2011	04‐May‐2012	288	288
PH2011BW13^a^	M	Adult	34.8	91	84	8+	22‐July‐2011	11‐January‐2012	174	174
PH2014BW01^b^, ^c^	M	Adult	53.6	100	95	6	23‐July‐2014	19‐May‐2015	301	301
PH2014BW02^a^	M	Subadult	18.3	74	70	—	23‐July‐2014	02‐February‐2015	195	195
PH2016BW01^b^	M	Adult	50.9	110	101	6+	03‐July‐2016	04‐April‐2017	276	276
PH2016BW03^b^	F	Subadult	24.8	86	81	1+	03‐July‐2016	26‐January‐2017	208	195
PH2016BW04^b^	M	Adult	49.1	114	101	6+	03‐July‐2016	22‐March‐2017	263	263
PH2016BW06^b^	F	Subadult	25.9	86	84	1	03‐July‐2016	21‐January‐2017	203	203
PH2016BW09^b^	F	Adult	46.7	113	101	5+	04‐July‐2016	23‐February‐2017	235	205
PH2016BW10^b^	F	Subadult	40.0	100	92	4	04‐July‐2016	09‐February‐2017	221	217
PH2016BW11^b^	M	Adult	36.6	98	92	5+	04‐July‐2016	25‐February‐2017	237	237
PH2016BW12^b^	M	Adult	36.8	103	93	6+	04‐July‐2016	06‐January‐2017	187	180
PH16BRW‐120350^c^, ^d^	M	Adult	51.6	112	124	8+	03‐July‐2016	06‐April‐2017	278	258
PH16BRW‐120353^c^, ^d^	M	Adult	51.6	112	105	7+	03‐July‐2016	31‐January‐2017	213	178

Note

These seals reported location data beyond December 31 of the tagging year. Duration between the first and last location is presented as *elapsed days*, while *CRAWL days* denote the total number of days for which the CRAWL movement model estimated the seal's location with a standard error of <25 km. The complete list of captured seals is recorded in Appendix A.

^a^ SPLASH tag without dive‐behavior time series.

^b^ SPLASH tag with dive‐behavior time series.

^c^ 24 × 1 hr % dry (haul‐out) data were unavailable.

^d^ CTD tag with dive‐behavior time series.

Satellite transmitters (hereafter “tags”) provided location and dive data for each seal using the Argos System (Harris et al., 1990). Most seals were instrumented with one primary and one secondary tag, and the data from each were combined into a single tracking time series. While secondary tags were expected to reveal haul‐out locations for up to 2 years, only two of the secondary (SPOT) tags (*n* = 28) provided >1 year of data (Appendix A), so we limited this study to the first year of data collection. Because seasonal patterns were of primary interest, we also limited our analyses to seals with tags that provided data beyond December 31 of the deployment year (*n*_2011_ = 5, *n*_2014_ = 2, and *n*_2016_ = 10).

Of the 17 seals in this study, all but two were instrumented with SPLASH tags (Wildlife Computers; 7.6 × 5.6 × 3.2 cm; 125 g in air) as their primary tag, while the remaining two seals were instrumented with CTD tags (Sea Mammal Research Unit; 10.5 × 7 × 4 cm; 545 g in air). All primary tags were attached using 5‐min epoxy and/or cyanoacrylate adhesive to either the fur between the shoulder blades or on the head depending on the size of the seal. We anticipated that the primary tags would remain attached to the seals until shed during their annual molt the following spring—a duration of about ten months depending on tagging date. The primary tags provided data on movements, diving, and haul‐out behavior. The 2011 primary tags (SPLASH) provided summary statistics for dive duration and maximum dive depth (for dives ≥3.5 m deep) as histograms, summarizing 6‐hr time blocks. All primary tags deployed in 2016 recorded the start time, end time, and maximum depth (resolution = 0.5 m, ±1%) of each individual dive, where start and end times were detected by crossing a 1.0 m depth threshold, and they did not collect 6‐hr histogram summaries. The primary tags deployed in 2014 were mixed; all collected 6‐hr histograms and some recorded individual dives, but the dive end times relied on a saltwater sensor that was prone to incorrectly pool sequential dives when intervening surface events were not detected. Hence, we only analyzed dive‐behavior data collected from tags deployed in 2016. All seals were also instrumented with a secondary tag (Wildlife Computers SPOT; 2.0 × 2.0 × 8.3 cm; 50 g in air). All secondary tags were permanently affixed to the rear flipper by screwing into a backing‐plate through two holes punched in the interdigital webbing.

Using R statistical software v3.4.2 (R Core Team, 2017), we applied a continuous‐time correlated random walk model (R‐package CRAWL v2.0.1; Johnson, London, Lea, & Durban, 2008) to estimate locations every 6 hr based on the tracking time series. Before applying the CRAWL model, we excluded implausible Argos locations, such as those that were on land or that failed to meet criteria for movement rates and turning angles (Appendix B). For the CRAWL analysis, we converted the locations to a Lambert's equal area map projection centered on the study area. Prior to further analysis, we excluded CRAWL location estimates that had standard errors >25 km (3.5% of all location estimates), which was commensurate with the spatial scale of the lowest‐resolution environmental data we used in our modeling. CRAWL locations were augmented with habitat metrics describing the distance to mainland (Wessel & Smith, 1996), bathymetry (National Geophysical Data Center, 2006), sea‐ice concentration (Cavalieri, Parkinson, Gloersen, & Zwally, 1996; Maslanik & Stroeve, 1999), and distance to the sea‐ice edge. The minimum distance to the ice edge was measured from the midday UTC (03:00 local time) location estimate to the nearest periphery of sea ice (≥15% concentration) composed of ≥10 contiguous 25 km pixels. Because there was a 294‐day gap between the higher spatial resolution AMSR‐E and AMSR‐2 sea‐ice data sets, we used the 25 km resolution SSM/I sea‐ice data in our analyses. Sea‐ice concentration was based on the average value within a 50 km radius circle (excluding land) centered on the midday location.

Diurnal and seasonal haul‐out behaviors were quantified using data from the primary tags, which binned daily summaries into 24 one‐hour increments. The tag reported the fraction (%) of each hour that its saltwater sensor was wet or dry (sampled at 10 s intervals). We defined hours that were ≥80% dry as “haul‐out” hours. The 80% threshold was robust because the distribution of hourly percent‐dry values was strongly bimodal; with 95% of all sampled haul‐out hours (*n* = 62,279) being either ≥80% dry (11.5%) or ≤25% dry (83.5%). We excluded the first week of post‐deployment behavior data prior to analysis to reduce potential biases associated with capturing seals close to shore.

Dive‐behavior analyses were based on data retrieved from the primary tags deployed in 2016 (*n* = 10) and included parameters for dive duration, dive depth (maximum), and surface duration. We temporally paired the dive metrics with the nearest 6‐hr CRAWL location and associated environmental attributes (e.g., ocean depth and sea ice). Dives were classified as bottom dives when the maximum dive depth was ≥75% of the mapped ocean depth. We did not attempt to classify bottom‐dives in shallow water (<10 m) where relationships between dive depths and water depths become increasingly uncertain due to inaccuracies in both the location and bathymetry data.

By comparing successive dive depths and intervening surface intervals (Appendix C), we classified behavior as: (a) *resting*, when the surface interval between successive dives exceeded 10 min; (b) *repetitive diving*, when ≥5 sequential dives attain maximum depths within ±15% of either of the two preceding dives —single dives >15% different were allowable within a repetitive‐diving episode; and (c) *mixed diving*, for all remaining dives not classified as repetitive.

We partitioned movement, dive, and haul‐out data into two habitat types: the shallow continental shelf (<300 m, *n* = 3,933 tracking days) and the deeper Arctic Basin (>1,000 m, *n* = 127 days). Seal locations over the steep shelf‐break (300–1,000 m, *n* = 23 days) were excluded from our dive‐behavior analyses to avoid highly misrepresentative relationships between dive depth and bottom depth that could arise from modest spatiotemporal mismatches between dive and location data.

Monthly dive summaries included the daily average time spent diving and the proportions of repetitive versus mixed diving. For each month, July–April, we estimated the average daily hours spent diving using the dive‐behavior time series of individual dives and surface times, and the 1 × 24 hr haul‐out classifications, both from the 2016 SPLASH‐tag deployments (*n* = 8). For each month and seal, we tallied the number of observed hours hauled out as well as the total number of observed hours that were sampled, and we used the resulting ratio to extrapolate an estimate of the total number of hours hauled out during a respective month. The remaining hours (not hauled out) were then allocated to three behavior classes based on proportions derived from the dive‐behavior time series. We considered the dive‐behavior data to be a representative sample of the time seals spent diving or resting at the surface during the hours they were not hauled out (Appendix D). Surface times >10 min in duration were assigned to “resting” only if the respective period did not overlap with a haul‐out hour. If it did overlap, then that surface interval was discarded since it had already been tallied into the haul‐out hours above. Dive sequences (including intervening surface times) were classified as “repetitive” or “mixed” as described earlier. For each month and seal, we tallied the total amount of time spent diving (repetitive and mixed) versus resting and used that ratio to allocate the remaining monthly hours “not hauled out” as either engaged in diving or resting. Average daily estimates of time spent diving were calculated by dividing the extrapolated monthly total of hours diving by the number of days in the respective month. We excluded seal months if the average distance to mainland was <5 km, because ocean depths near shore were often less than the tag's designated 3 m threshold for dive detection.

To inform our interpretation of ringed seal movements, habitat use, and haul‐out behavior, we built five model sets targeting these response variables (Appendix E): I. Movement Rate, II. Distance to Mainland, III. Distance to Ice‐Edge, IV. Ice Concentration, and V. Haul‐Out Time. Each response variable was modeled with respect to four independent factors: *Sex* (MALE vs. FEMALE), *AgeClass* (ADULT vs. SUBADULT), *Season* (OPEN‐WATER vs. ICE‐COVERED), and *CapYear* (2011/2014.16). *Season* refers to the general timing of the open‐water and ice‐covered periods. The ICE‐COVERED season, which occurs from December through June was characterized by the advance of sea‐ice south into the Bering Sea where it would remain until spring when it would begin to retreat to the north. The OPEN‐WATER season occurs from July through November, and is characterized by generally ice‐free waters over the continental shelf of the entire Bering Sea and much of the Chukchi Sea. The factor *CapYear* was included to assess whether a disease outbreak that began in 2011 may have influenced ringed seal movements and/or behavior. Ultimately designated as an “Unusual Mortality Event” (UME) (NOAA, 2011a, 2011b, 2012, 2014; Stimmelmayr et al., 2013), none of the 2011 seals included in our analyses showed obvious symptoms (e.g., alopecia, lethargy, skin inflammation, or unusual molting patterns). However, several other seals captured in 2011 were symptomatic. In contrast, during both the 2014 and 2016 field seasons, no seals were observed to be symptomatic.

All models were constructed using R Statistical Software. Prior to modeling, we removed the first week of data from each seal's time series to reduce any influence of capture and handling. Three response variables: *Movement Rate*, *Distance to Mainland*, and *Distance to Ice Edge* (model sets I–III), were square root transformed prior to analysis and were modeled using linear mixed effects models (R‐package *nlme* v. 3.1‐140, Pinheiro, Bates, DebRoy, & Sarkar, 2019) assuming a normal distribution and identity link function. To account for spatial autocorrelation, we employed a first‐order autoregressive (AR1) structure. Because the data for *Ice Concentration* in model set IV was proportional (i.e., range = 0–1), we used generalized linear mixed models (R‐package *glmmTMB*; Brooks et al., 2017) with a beta distribution and logit link. We transformed the data following Smithson and Verkuilen (2006) to address zeros and ones. To understand the use of sea ice when it was generally available, we partitioned the data for model set IV by *Season*, and developed models for the ice‐covered period only. *Haul‐out Time* (model set V) was also analyzed with R‐package *glmmTMB*, but using a Poisson distribution and log link. In this model set, we were interested in understanding the factors associated with time spent when haul‐out occurred (not whether haul‐out occurred), and so we filtered our data to include only those days with ≥1 hr spent hauled out. As such, our models did not require adjustments that would otherwise be needed for zero‐inflation. Finally, all models included random effects to account for individual variability among seals.

For each model set, we followed a systematic model selection procedure. First, we generated all possible single‐ and multivariate mixed models. We assessed model performance based on parsimony (using Akaike's information criterion; AIC_C_) and then modified the highest performing models (ΔAIC_C_ <2) by adding two‐way interaction terms. The full model set was then re‐assessed and ranked. Models within each set having the lowest AIC_C_ were considered “best,” though other models with ΔAIC_C_ within 2.0 of the highest‐ranking model were also deemed comparable (Burnham & Anderson, 2002). Visual inspections of residual plots from all significant models revealed no obvious deviations from homoscedasticity (Zuur, Ieno, Walker, Saveliev, & Smith, 2009). Finally, we used R‐package *emmeans* (Lenth, 2019) to estimate marginal mean values from the best models (Appendix F) and to make multiple comparisons because this method is useful for summarizing the effects of factors when subjects are repeatedly measured and have unequal sample sizes (Lenth, 2016).

## 3 RESULTS

Of the 39 ringed seals captured and tagged (Appendix A), a total of 17 (Table 1) met the criteria for tag longevity in order to be included in our analyses. These 17 ringed seals included 12 adults (9 ♂, 3 ♀) and 5 subadults (2 ♂, 3 ♀). Mean body length was 102.5 cm (*SD* = 9.3) for adults and 88.2 cm (*SD* = 10.0) for subadults. The mean weight of adults was 40.7 kg (*SD* = 11.2) and subadults was 26.8 kg (*SD* = 8.0). The mean length ($\bar{x}$ = 92 cm, *SD* = 0.8) and weight ($\bar{x}$ = 28.0 kg, *SD* = 4.8) of adult seals tagged in 2011 (*n* = 4) were both significantly less than the mean length ($\bar{x}$ = 107.8 cm, *SD* = 6.4) and weight ($\bar{x}$ = 47.1 kg, *SD* = 6.7) of adults tagged in 2014 and 2016 (*n* = 8).

A total of 52,431 satellite locations were received; the median number of locations per seal was 2,778 (range = 2,146–6,020) and the median tracking duration was 239 days (range = 178–331). The median time interval between sequential locations was 0.52 hr (range = 0.01–1,157), with few long temporal gaps (99th percentile = 20.3 hr). Higher‐quality locations (Argos classes = 1, 2, and 3) comprised 7,471 (7.2%) of the seal locations. Filtering excluded 3,757 lower‐quality locations (Argos classes = 0, A, B, or Z). After applying the CRAWL model and excluding 596 estimated locations with SEs >25 km, the final data set contained 16,260 location estimates, representing 4,083 individual seal tracking days (median = 237 tracking days/seal; range = 174–330). During the period when all 17 seals were tracked (August to December) the median cumulative distance traveled was 4,790 km/seal (range = 2,719–5,988) (Figure 3d).

**FIGURE 2:**
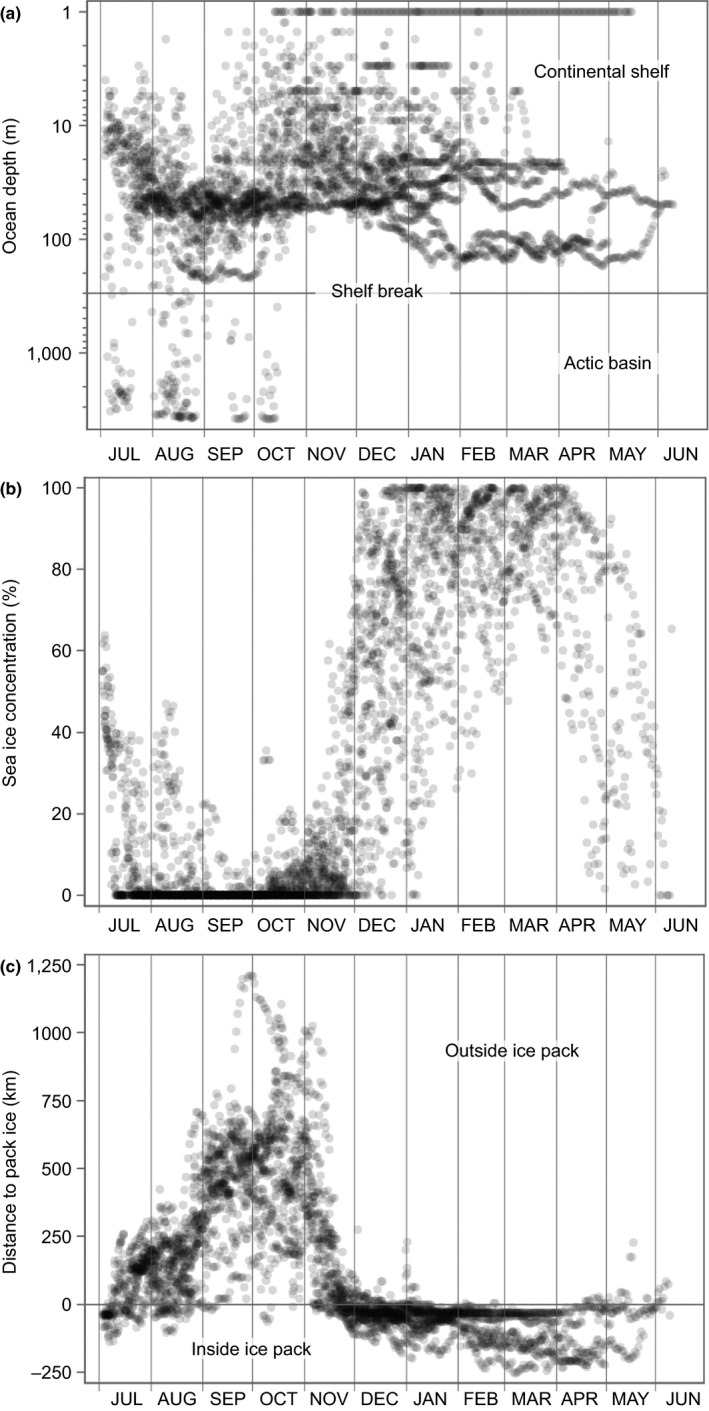
Seasonal time series of daily ringed seal habitat attributes: (a) ocean depth; (b) mean sea‐ice concentration (within a 50 km radius); and (c) distance to the edge of the pack‐ice—where negative values represent distances from inside the ice pack. Ocean depth (a) is shown on a log scale with a horizontal line at the shelf‐break (300 m depth)

**FIGURE 3:**
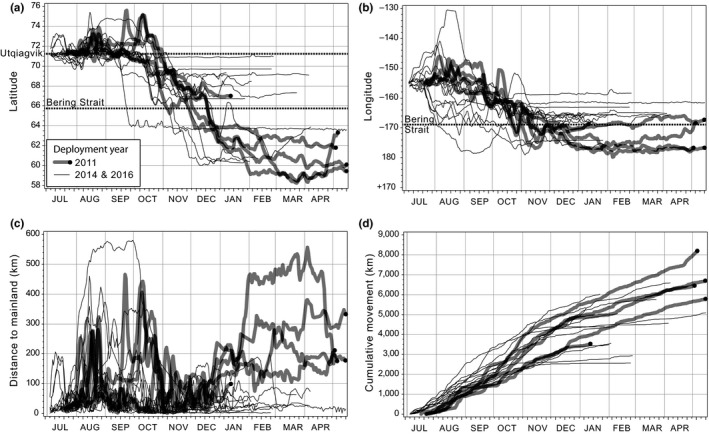
Seasonal movements of 17 ringed seals with respect to (a) latitude, (b) longitude, (c) distance to mainland (islands excluded), and (d) cumulative daily tracking distance. Thin black lines are seals tagged in 2014 and 2016. Thick gray lines with black terminal dots are seals tagged in 2011. Distances calculated based on daily CRAWL location estimates. Seventeen seals provided data through January, and then, sample size declined to 12, 8, 7, 5, and 2 seals during February through June, respectively

Ringed seals occupied continental shelf waters on >96% of the tracking days, with some seals making off‐shelf forays into the Arctic Basin in July to October (Figure 1b). All ringed seals occupied waters with sea ice during winter, but most occupied open‐water south of the pack‐ice during September–October (Figure 2). With one notably early southward movement into the Bering Sea in mid‐September, all seals had moved south of Utqiaġvik by early November. Some seals continued to move during winter while others occupied specific locales for extended periods. Eight seals (6 adults, 2 subadults) moved south through the Bering Strait from November to mid‐December, while nine others (7 adults, 2 subadults) remained in the Chukchi Sea into January (Figure 3a). Of the four 2011 seals that wintered in the Bering Sea, three moved to deeper waters south of the Gulf of Anadyr (Figures 1b and 1, 3b), while the fourth 2011 seal and all four of the 2016 seals that wintered in the Bering Sea moved south to waters near the Yukon‐Kuskokwim Delta (Figures 1b and 1, 3b). There was high seasonal variability in distance to mainland during both the ice‐covered (Jan to Jun) and open‐water (Jul to Dec) seasons (Figure 3c). From November to January, distance to mainland was generally less and not as variable because many seals were near the Bering Strait (Figure 3c). Six of 10 seals tagged in 2016 remained in the Chukchi Sea for the duration of winter tracking—four along the Alaskan coast between Utqiaġvik and Cape Lisburne, one at the mouth of Kotzebue Sound, and one at Kolyuchin Inlet in northern Chukotka. One 2014 seal moved into the western Chukchi Sea during winter, just north of the coast of Chukotka; and one 2011 seal was near the Bering Strait when its tag stopped transmitting in February 2012.

**FIGURE 4:**
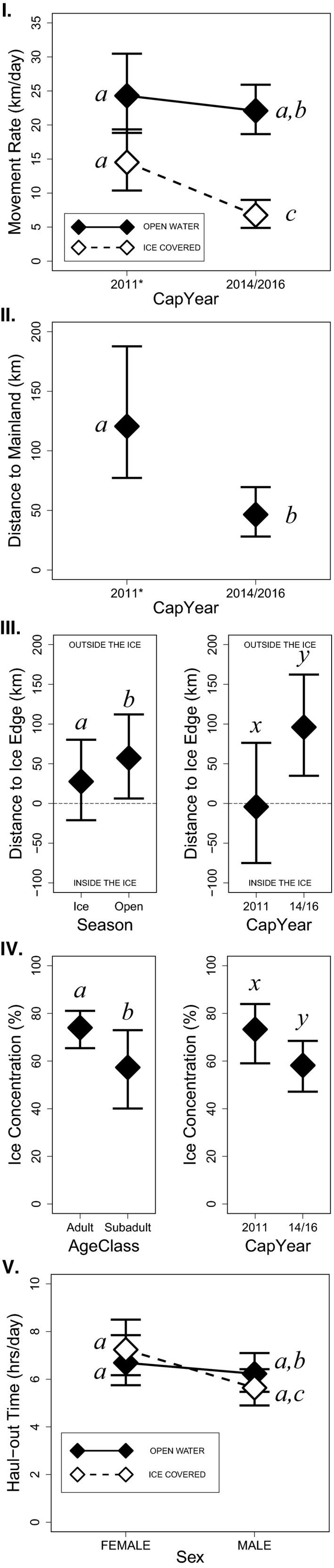
Marginal means [diamonds] with 95% CI for models of (I) movement rate, (II) distance to mainland, (III) distance to ice edge, (IV) concentration of ice occupied, and (V) haul‐out time. Figures show the estimated marginal means generated from the best model from each of the five model sets (Table 3; Appendix E). The factor *Season* is designated as OPEN‐WATER (Jul–Nov) and ICE‐COVERED (Dec–Jun). The factor *CapYear* is designated according to year of tag deployment (2011 vs. 2014/16). The factor *AgeClass* is designated as ADULT and SUBADULT, and *Sex* is as FEMALE and MALE. Negative values in panel III—*Distance to Ice Edge (km)* refer to distances from inside the pack ice to the ice edge, and positive values from outside the pack ice to the ice edge. Note that panels I and V depict interactions, panel II depicts a univariate model, and panels III and IV depict bivariate models. *Year of Unusual Mortality Event

Ringed seal movements, spatial use, and habitat attributes varied over yearly and seasonal scales, and with respect to demographic variables (Figure 4; Appendix F). Movement rate was best described by a model with an interaction between *Season* and *CapYear* (i.e., year of tag deployment), with the greatest rates occurring during the open‐water season and also the 2011 ice‐covered season. A univariate model that included *CapYear* best described distance to mainland, with seals in 2011 occupying areas significantly farther offshore (120.6 km vs. 46.5 km; Appendix F). The distance to the ice edge was best explained by a bivariate model that included *Season* and *CapYear*, both of which were significant. As expected, the presence of ice negatively influenced the distance from the sea‐ice edge, with seals remaining closer to and deeper within the pack‐ice during the ice‐covered season. Meanwhile, the seals tagged in 2011 tended to stay closer to and deeper within the pack‐ice than seals tagged in 2014 or 2016. The concentration of sea ice occupied was best explained by a bivariate model that included *AgeClass* and *CapYear*, but both factors fell short of statistical significance (*p* = .060 and .067 respectively). Finally, the time spent hauled out on the ice was best explained by a model in which *Sex* and *Season* interacted. During the ice‐covered season, males spent less time hauled out (on days with ≥1 haul‐out hour) than during the open‐water season (*p* = .026). Though not statistically significant (*p* = .087), the largest difference in marginal mean haul‐out hours was between males and females during the ice‐covered season.

**FIGURE 5:**
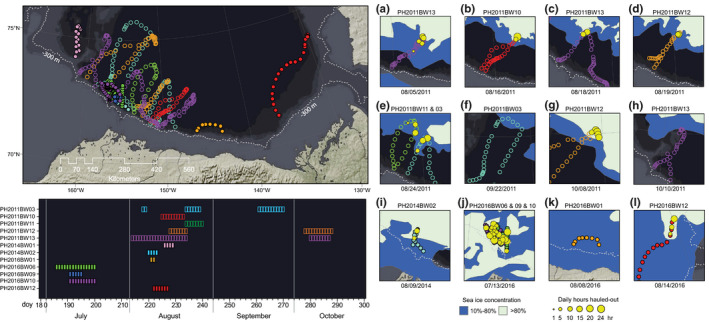
Off‐shelf forays into deeper water. In all panels, seals instrumented in 2011 are distinguished by open symbols, and those in 2014 and 2016 by solid symbols. *Upper left*—Locations (4 per day) of 12 ringed seals when occupying waters >1,000 m deep, colored by individual seal. *Lower left*—Rectangles indicate dates spent off‐shelf, with the same colors as the locations shown above. *Right*—Distribution of sea‐ice and haul‐out behavior during forays into waters >1,000 m deep (*n* = 11 seals). Days when one or more haul‐out hours were recorded are overlaid as yellow dots scaled in size by the total hours hauled out that day. Sea‐ice conditions (U.S. National Ice Center, 2019) on the date (shown below each panel) correspond temporally with the more northerly locations and show two classes of ice concentration: marginal (blue, 10%–80%) and contiguous (light blue, >80%)

**FIGURE 6:**
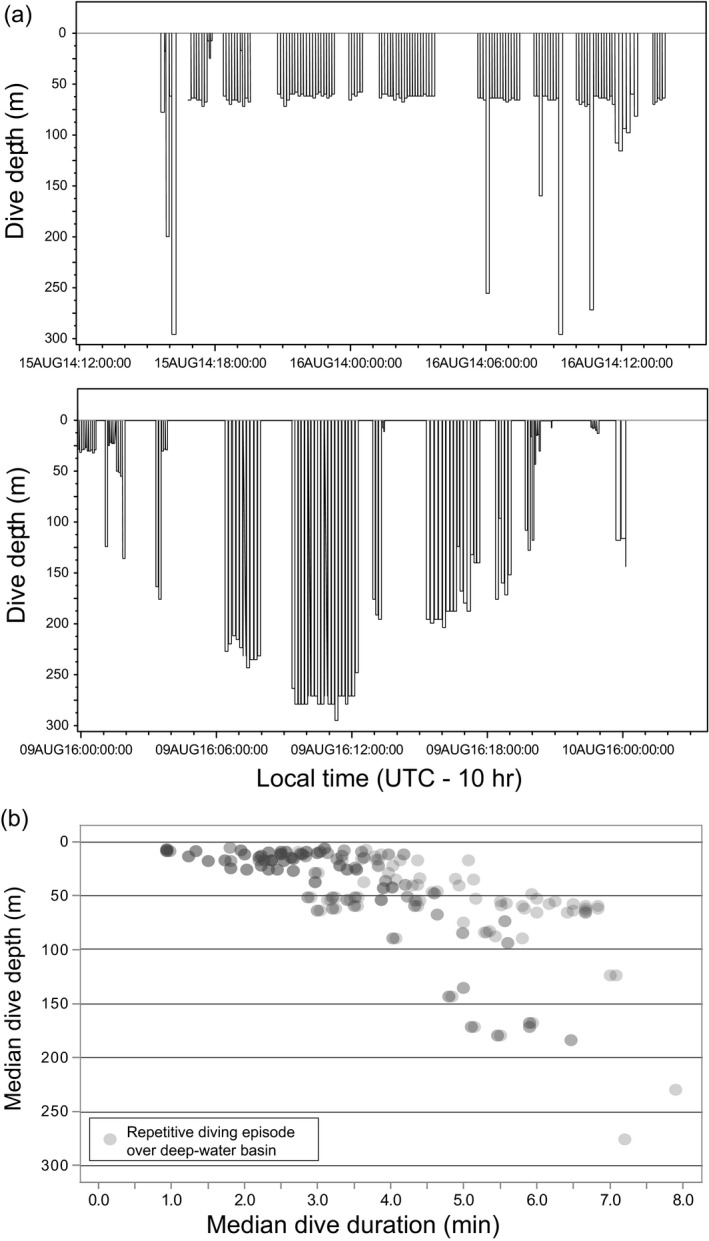
*Top*—Example of repeated diving to ~60 m depth by one seal during one day while occupying the deep‐water Arctic Basin. Note the occasional exploratory dives to 200–300 m depths. *Center*—Example of relatively deep repetitive dives in the Arctic Basin (by one seal during one day) suggesting that prey may be present in deeper strata, and supporting the notion that exploratory dives can have payoffs. *Bottom*—Records of repetitive‐diving episodes in the Arctic Basin. Relatively few repetitive dive episodes occurred at depths >100 m (max ~275 m; shown in *Center* panel)

From July to mid‐October, 12 of 17 tagged seals (71%) undertook forays into the deep Arctic Basin (Figure 5; Appendix G): including 7 males (2 subadult) and 5 females (1 subadult). All 2011 seals (*n* = 5) ventured into the Arctic Basin, contributing 9 of the 16 observed forays. The median duration of the forays was 7 days (range = 2–21) (Figure 5). While in the presence of sea ice in the Arctic Basin, seals tended to haul out for extended periods of time (median duration = 11 hr, maximum duration = 34 hr, *n* = 42; Appendix G)—usually returning directly thereafter to the continental shelf. Three seals made a second foray off‐shelf to the ice edge (Figure 5). During three other forays, seals failed to encounter substantive ice cover and haul‐outs were not recorded (Appendix G). Diving behavior in the Arctic Basin consisted of both mixed‐ and repetitive dives, with the latter comprising 40% of the recorded dives (*n* = 2,119). Repetitive dives were occasionally punctuated by intermittent deeper dives of 200–300 m (Figure 6); on occasion these deeper dives immediately preceded repetitive diving to deeper strata.

By December, all seals tended to occupy regions with high sea‐ice concentration on the continental shelf (Figure 2). By March and April, among seals that wintered in the Bering Sea (two adult females, one juvenile male, and three adult males), the juvenile and two adult males began making modest northward movements, but all remained south of the Bering Strait (Figure 3a). In April, the average sea‐ice concentration occupied by seals began to decrease (Figure 2b), suggesting that they were not aggressively pursuing the retreating ice edge northward. While we observed no statistically significant differences in the distance to ice edge based on sex or age class (Figure 4; Appendix F), we do note that, over the entirety of the year, adults tended to occupy higher concentrations of ice than subadults (79.98% vs. 57.32% respectively; *p* = .060). Those seals tagged in 2011 also appeared to occupy regions deeper within the pack‐ice than the seals tagged in 2014 or 2016 (Figure 4; Appendix F), though this was not significant (*p* = .067). No other differences in sea‐ice location or concentration based on sex or age class were observed.

**FIGURE 7:**
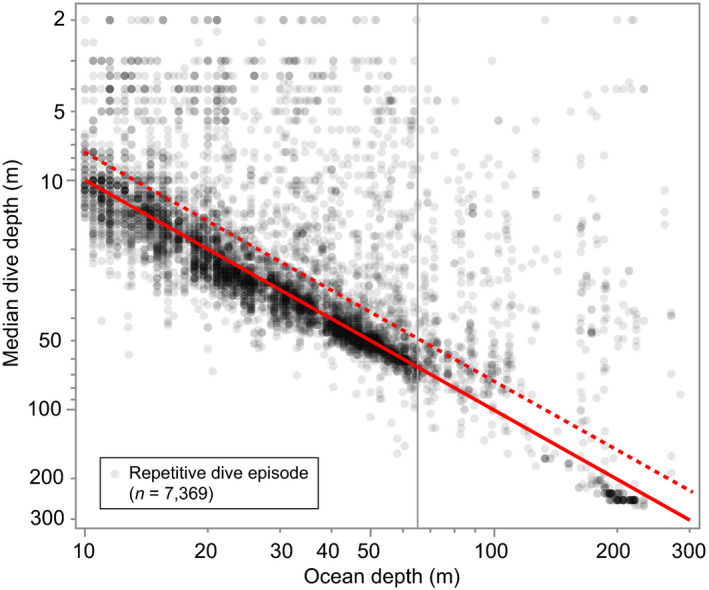
Median dive depth recorded during episodes of *repetitive diving* to similar depths in relation to the average ocean depth at locations on the day of the diving. Analysis was restricted to days when seals were located in water 10–300 m deep. The solid red line denotes a 1:1 dive depth to ocean depth relationship, and the dotted red line denotes the dive‐depth threshold for classification as *bottom diving*. The gray vertical line denotes the 65 m isobath which is demarcated in Figure 1 with the light gray shading. Note log scales on both axes. See Figure 8 for a summary of dive behavior based on the dive histogram data received from tags deployed in 2011. Dives that implausibly exceeded ocean depth were likely due to errors in the estimated seal locations, errors, or generalizations in the coarse‐resolution bathymetry data, or imprecision in assigning locations to dives

**FIGURE 8:**
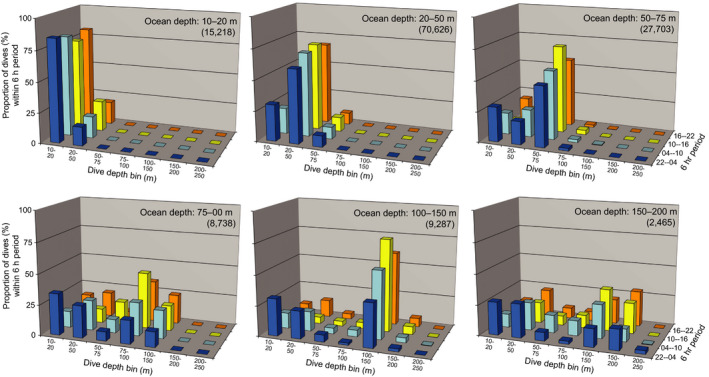
Dive histogram data corroborating daytime bottom‐diving behavior among the ringed seals tagged in 2011. SPLASH tags deployed on ringed seals in 2011 (*n* = 5) provided summarized “histogram” data containing the number of dives to ocean‐depth intervals during four 6‐hr periods (charted here in local time, UTC‐10 hr). We partitioned dive data into days when seals were located where the ocean depths were congruent with the six most commonly visited dive‐depth bins and charted the relative proportion of dives in each depth bin, for each 6‐hr period. Numbers in parentheses are the number of dives summarized in the respective chart. Results corroborate that most dives attained depths near the ocean bottom (as in Figure 7) and that deeper diving was more common during the midday (10:00–16:00) hours (as in Figure 10)

**FIGURE 9:**
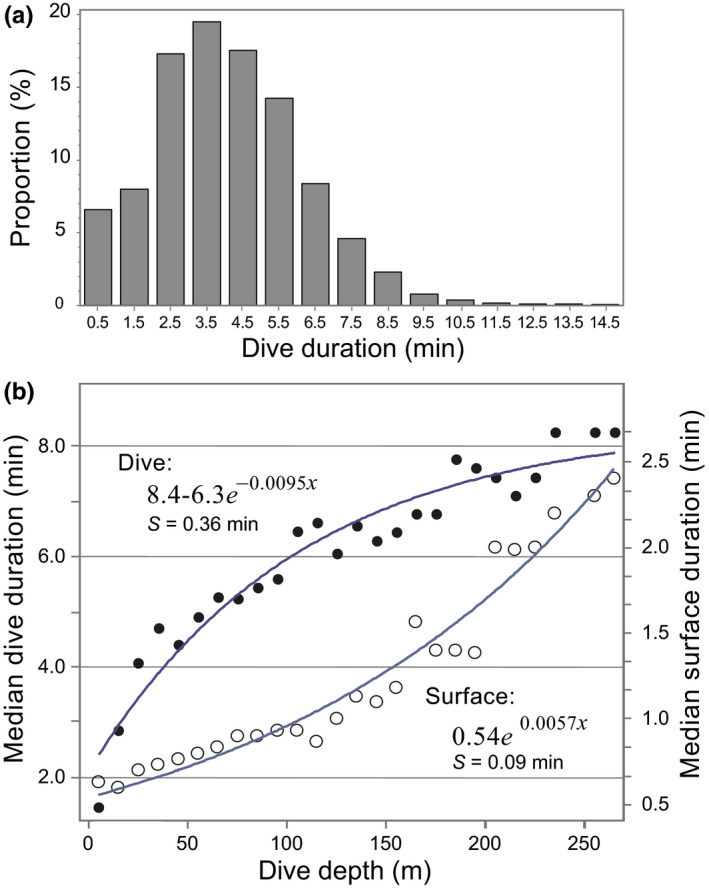
(a) Dive duration frequency distribution across 1‐min duration bins. (b) Logarithmic regressions fitted to median dive duration (solid circles) and median surface duration (open circles) as a function of median dive depth in 10‐m depth intervals (using only intervals with *n* ≥ 10 dives). Standard error of the regression (S) represents the average distance (minutes) that the medians fall from the regression line. Analysis used the dive‐behavior time series collected by SPLASH and CTD tags deployed in 2016 (*n* = 10), for dives ≤15 min in duration (*n* = 81,916)

The daily activity budget was dominated by diving ($\bar{X}$ = 16.5 hr/day); with most of that time ($\bar{X}$ = 13.2 hr/day) spent repetitive diving (Table 2). Other than in July, which showed the lowest monthly mean, the proportion of daily hours spent diving remained relatively constant. The proportion of repetitive dives was consistent from July to January (~80%) but decreased in February to 55%, though by this time the sample size had declined to two adult males. Bottom‐dives comprised 65% of all dives recorded on the continental shelf (*n* = 96,414). During 7,369 episodes of repetitive diving (consisting of 67,355 dives), 78% met the criteria for bottom‐dives (Figure 7). Dive histogram data from seals tagged in 2011 also indicated that most dives over the continental shelf were bottom‐dives (Figure 8). The median dive duration was 3.9 min (99th percentile = 10.7 min, *n* = 81,916). Approximately 67% of all dives recorded ranged from 2.5 to 5.5 min (Figure 9a). The median surface duration between dives was 0.7 min (~42 s) (99th percentile = 4.1 min, *n* = 76,964). With increasing dive depth (up to 270 m), dive duration increased asymptotically while the intervening surface time increased exponentially (Figure 9b). The regressions of dive and surface times in Figure 9b used the medians of 10‐m dive‐depth bins; however, it should be noted that the maximum dive durations in each of those bins were consistently 10–12 min—which may represent the physiological dive‐duration limit for ringed seals (Lydersen, Ryg, Hammill, & O'Brien, 1992).

**TABLE 2:** Monthly estimates of the mean hours per day spent diving, and the proportion (%) of those hours spent engaged in episodes of repetitive diving to similar depths

Month	*n*	Diving (h)		Repetitive (%)		Sample (%)	
		Mean	*SD*	Mean	*SD*	Mean	*SD*
July	8	14.7	2.0	80.0	15.5	19.2	7.2
August	7	17.1	2.0	80.2	4.0	34.1	10.8
September	7	17.3	3.3	81.1	10.0	24.8	10.8
October	7	17.4	5.1	81.8	9.3	32.2	14.6
November	6	16.4	2.3	81.3	12.9	29.0	11.5
December	6	16.5	3.0	82.5	8.8	30.7	12.3
January	5	16.2	3.9	80.1	9.3	18.4	3.5
February	2	16.3	0.5	54.6	16.4	15.8	3.1
Pooled	48	16.5	3.1	79.9	11.4	26.5	11.6

Note

Sample (%) is the fraction of the month for which we obtained dive‐behavior time series data for any given seal month. For each month, at least a 10% sample of the dive‐behavior time series data was required for a seal to be included in the respective monthly estimate (“*n*” is a seal month). Analysis used the 8 SPLASH tags deployed in 2016. Seal months with an average distance from the coast of <5 km were excluded (*n* = 4 seal months).

**FIGURE 10:**
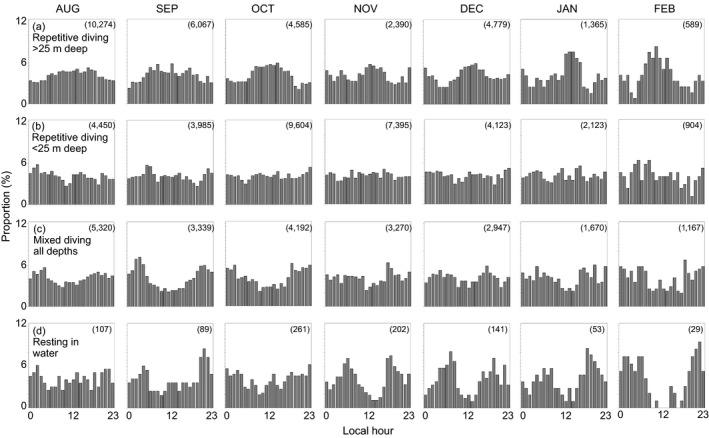
Proportion of dive‐behavior classifications that occurred in each hour of the day (local, UTC‐10 hr) in each of 7 months. Episodes of repetitive diving during which median dive depth was (a) >25 m or (b) ≤25 m deep; (c) episodes of diving to mixed depths, and (d) periods of resting at the surface for >10 min while also unassociated with haul out. Sample sizes (number of dives or resting periods) are shown in the upper right of each panel. Data are summarized from the dive‐behavior time series collected from SPLASH tags deployed in 2016 (*n* = 8 ringed seals)

Monthly diurnal frequency distributions of dive‐behavior observations that were classified into each of four behavior classes revealed daily patterns that changed seasonally (Figure 10). For example, there was a higher frequency of repetitive dives during midday for depths >25 m only (Figure 10a,b)—becoming increasingly prevalent as day‐length diminished from late summer into autumn and winter. The shape and magnitude of the mixed‐dive histograms (Figure 10c) somewhat resembled the resting histograms, but with less‐well defined diel and monthly patterns. Resting behavior exhibited diel and monthly patterns that were somewhat complementary to those observed for repetitive dives >25 m deep (Figure 10d).

Hourly percent‐dry time series data were obtained for an average of 72% (*SD* = 8.9%) of the tracking period. After excluding data from the first week of satellite tag deployment and data that were collected off‐shelf, the median duration of uninterrupted haul‐out bouts was 3 hr (range 1–28, *n* = 1,025 haul‐outs). Time spent hauled out per day was significantly longer during the open‐water than during the ice‐covered season for males (6.23 vs. 5.64 hr/day) (Figure 4; Appendix F). There was no significant difference in the daily time spent hauled out between seals tagged in 2011 and those tagged in 2014 and 2016. Terrestrial haul‐out behavior was frequently observed among untagged ringed seals near Utqiaġvik in the summer of 2011—a number of which were captured and tagged (Appendix A). Observations of terrestrial haul‐outs are extremely rare for ringed seals in Alaska (North Slope Borough, *unpublished data*) and no such behavior was documented in 2014 or 2016. Also, during 2014 and 2016 on days with a CRAWL location estimate, 86% of the haul‐out hours occurred >10 km from the coast.

**FIGURE 11:**
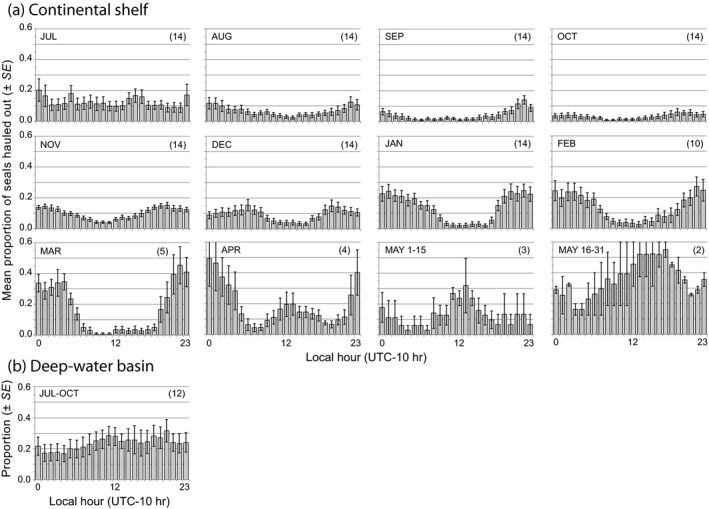
Monthly diurnal haul‐out behavior shown as the mean proportion (±*SE*) of ringed seals hauled out during each local hour (UTC‐10 hr) when seals were (a) over the continental shelf, and (b) during forays into the deep‐water Arctic Basin. Monthly sample sizes (*n* seals) are shown in parentheses. May is split into two periods. Data obtained during deep‐water forays (July–October) were pooled to bolster sample size

The proportion of tagged seals hauled out exhibited patterns that varied both diurnally and monthly (Figure 11). In July, 15%–20% of the seals were hauled out during any given hour of the day with little indication of a diel pattern. The proportion of seals hauled out declined from August through October, with a subtle indication of nocturnal preference. From November through March, the proportion of seals that hauled out nocturnally increased. Haul‐out behavior switched from nocturnal to diurnal in April and May as seals showed a strong midday preference; however, by April to late May the sample size had declined to two adult males.

## 4 DISCUSSION

Ringed seals instrumented with satellite transmitters near Utqiaġvik, Alaska provided movement and dive‐behavior data that both corroborated and expanded prior knowledge. The ringed seals in our study migrated to the southern Chukchi and Bering seas for winter, like those tagged by Crawford, Frost, et al. (2012) near Kotzebue, Alaska, and those tagged by Harwood et al. (2012) near the entrance of Amundsen Gulf, Canada. Data from tens of thousands of individual dives provided new insights into ringed seal foraging behavior, notably a propensity to repeatedly dive to depths at or near the ocean floor. Unique to this study were several brief mid‐ and late‐summer movements into the deep‐water Arctic Basin, where seals spent most of their time hauled out on the available pack‐ice. Though not statistically significant, we provide some evidence of demographic segregation in sea‐ice concentration between adult and subadult seals as observed by Crawford, Frost, et al. (2012). However, other factors may also have been involved. For example, in the winter of 2011–2012, the sea‐ice maximum extent (in March) in the Bering Sea was the largest ever recorded in the satellite record (since 1979), as was the average sea‐ice concentration (77%) (Fetterer, Knowles, Meier, Savoie, & Windnagel, 2017). By comparison, average sea‐ice concentration in March in the Bering Sea was 64% in 2015 and 68% in 2017. Further, the seals tagged in 2011 displayed physical traits and spatial distributions that were consistent with the purported existence of a pelagic ringed seal ecotype. In the sections that follow, we expand upon these topics and offer speculations about how ringed seal movements, energy requirements, and physiological states may have interacted to shape the behaviors we observed.

Ringed seals tagged near Utqiaġvik almost exclusively utilized continental shelf habitat in the Chukchi and Bering seas (Figure 1). All seals traveled extensively during autumn, covering vast cumulative distances (see Harwood et al., 2012) on migrations that lasted until early winter (Figure 3d). During winter, movements were restricted for some seals, extensive for others, and habitats occupied were varied and widely distributed (from 57°N to 70° N latitude). Some seals stayed close to the coast in relatively shallow water, even stopping and maintaining a localized winter residency (Figures 1 and 3), while others went far offshore into the Bering Sea and moved continuously all winter in the dynamic pack‐ice. Diversity in behavior and of habitats occupied suggests that, as a species, ringed seals can exploit a breadth of niches. We found some evidence of demographic habitat partitioning (Figure 4; Appendix F). Adults appeared to occupy winter habitats with higher sea‐ice concentration, suggesting that different reproductive and life‐history states (e.g., mating adults vs. growing subadults) may lead to different habitat requirements Crawford, Frost, et al. (2012). Adult and subadult ringed seals tagged by Crawford, Frost, et al. (2012) near Kotzebue, Alaska, wintered in distinctly different regions, with subadults moving farther south into the Bering Sea, while adults stayed primarily in the southern Chukchi Sea. Though we also noted evidence suggesting demographic differences in habitat use (Figure 4; Appendix F), our results were not statistically significant. Our results indicate, however, that year of tag deployment was important to understanding the movements of ringed seals (Figures 3 and 4), which may be important in light of the UME that began in 2011.

Most of the seals in our study (71%) made brief (~week long) off‐shelf forays during summer that appeared to be deliberate and sometimes far‐ranging efforts to reach the retreating sea ice (Figure 5, Appendix G). Broad‐scale movements by ringed seals during the open‐water season are not unprecedented, such as populations in Svalbard that make long distance movements to seasonally access productive habitats (Freitas, Kovacs, Ims, Fedak, & Lydersen, 2008; Hamilton, Lydersen, Ims, & Kovacs, 2015). Off‐shelf movements by Utqiaġvik seals were notable because they apparently abandoned more productive continental shelf habitat (Born, Teilmann, Acquarone, & Riget, 2004; Kingsley, Stirling, & Calvert, 1985; Teilmann, Born, & Acquarone, 1999) in favor of deep‐water Arctic Basin habitat of generally lower quality (Frey et al., 2016). Given their dive‐depth constraints (Lydersen et al., 1992), ringed seals that forage in deep water may have limited access to prey or incur higher foraging costs (Hamilton et al., 2015). Upon reaching the sea ice in the Arctic Basin, ringed seals spent more time hauled out than foraging. That 25% of these seals returned for a second time suggests potential benefits that may result from this behavior. This apparent motivation to haul out may reveal physiological constraints, such as those relating to the energetics of their molt (Crawford, Vagle, & Carmack, 2012; Majewski et al., 2016).

Distinct patterns in the dive data suggest that the ringed seals in our study frequently engaged in focused bouts of repetitive diving, the attributes of which are suggestive of active foraging behavior. Specifically, tagged seals repeatedly dove to near‐constant depths, showed near‐constant dive durations and intervening surface times (Appendix C), and exhibited this behavior during substantial portions of the day (Table 2). Focused foraging behaviors can maximize energetic profitability when they result in repeated capture and consumption of aggregated prey—a strategy that makes energetic sense in patchy environments (Schoener, 1971). Repetitive diving also occurred more frequently during midday, when ambient light is brightest (Figure 10), and was spatially allocated in favor of habitats where prey species are known to aggregate—that is, the continental shelf seafloor (Benoit, Simard, Gagné, Geoffroy, & Fortier, 2010). If repetitive‐diving bouts are indeed indicative of active foraging efforts, then their prevalence in the data show that ringed seals forage on average >12 hr/day from August through January (Table 2).

The tendency for most repetitive‐diving bouts to occur at or near the seafloor (Figure 7) may be related to the ecology of their prey. Ringed seals prey upon zooplankton (Lowry et al., 1980) and planktivorous fish (Crawford et al., 2018), both of which make synchronous diel vertical migrations (DVM) into deeper waters during the brightest hours of the day (Hays, 2003; Rabindranath et al., 2011; Stich & Lampert, 1981)—but, as potential prey themselves, face trade‐offs between their own metabolic needs and predation risk (Pearre, 2003). Among Arctic cod (*Boreogadus saida*), which are an important forage species for ringed seals (Holst, Stirling, & Hobson, 2001), the larger and more energy‐rich adults have greater metabolic stores and lower food limitation that enables them to remain longer at depth—decoupling them from closely following the DVM of zooplankton into shallower water where predation risk is higher (Benoit et al., 2010). Dense aggregations of adult cod that form in the demersal zone can physically displace smaller conspecifics into shallower water (Benoit et al., 2010; David et al., 2016; Farley et al., 2017). Thus, Arctic cod physiology and behavior may set up an energetic trade‐off in which ringed seal forage resources are partitioned by prey body‐size (i.e., benefits to seals) and prey depth (i.e., cost to seals). Repetitive diving to the bottom may thus reflect optimal foraging (Waddington & Holden, 1979) in which larger and more energy‐rich cod are targeted (Bowen, Tully, Boness, Bulheier, & Marshall, 2002). This behavior would be consistent with an energy maximization strategy (Bergman, Fryxell, Gates, & Fortin, 2001; Santini & Chelazzi, 1996) that invests more energy into deeper or longer dives to achieve a higher net energetic intake rate than would be possible by foraging on more accessible but less energetically profitable prey. It may also partially explain the tendency for larger bodied seals to dive less frequently, but for longer durations (Crawford et al., 2018).

Repetitive diving occasionally occurred in the very deep waters of the Arctic Basin (Figure 6b). This behavior has been reported previously (Gjertz, Kovacs, Lydersen, & Wiig, 2000) and may be related to concentrations of primary productivity occurring in the upper water column during the late summer/early fall (Ardyna et al., 2013). Subsurface primary productivity is attractive to zooplankton and planktivorous fish (Crawford, Vagle, et al., 2012; Farley et al., 2017; Greenstreet et al., 2006; Majewski et al., 2016), potentially creating foraging patches that also attract ringed seals (Scott et al., 2010). Relatively shallow repetitive‐diving bouts over the deep‐water Arctic Basin were occasionally punctuated by single dives to substantially greater depths (Figure 6a). Perhaps exploratory in nature (Simpkins, Kelly, & Wartzok, 2001), these intermittent deep dives are consistent with a strategy of searching alternative foraging patches to minimize lost foraging opportunities (Kohlmann & Risenhoover, 1998; Lima, 1985), which may be more profitable in habitats with lower prey densities, heterogeneously distributed prey, or when a foraging patch is nearing depletion (McNair, 1983). Our observation of ringed seals shifting their repetitive‐diving behavior into deeper strata in the water column (Figure 6b) suggests that exploratory dives may have been profitable on occasion.

Temporal patterns of diving, resting at the surface, and hauling out (Figures 10 and 11) suggest that ringed seals modify their daily activities in response to ambient conditions and as an adjustment to the potentially high sensitivity of their prey to light (Berge et al., 2020). Repetitive diving to depths >25 m was more common during midday and became increasingly frequent at midday as day‐length diminished in winter. Repetitive dives <25 m deep did not show a diel or seasonal pattern. Although ambient light rapidly attenuates with water depth (Naik, D’Sa, Gomes, Goés, & Mouw, 2013), pinniped vision is well adapted to low‐light levels (Levenson & Schusterman, 1999). That ringed seals engaged in deeper foraging dives more often during midday, especially during the dark winter, suggests that visual hunting tactics may be important to foraging success (Hanke, Wieskotten, Marshall, & Dehnhardt, 2013).

Resting and haul‐out were more prevalent behaviors at night (Figures 10 and 11). We found that during onset of the ice‐covered season, seals hauled out more often during the darkest hours of the day (Figure 11), consistent with previously observed patterns of nocturnal haul‐out behavior in ringed seals (Crawford et al., 2018; Härkönen et al., 2008; Kelly et al., 2010). Furthermore, diurnal patterns from the binned dive data reported by tags deployed in 2011 were consistent with the aforementioned patterns that ringed seals dove most often to depths near the seafloor and during midday (Figure 8).

The relative value of habitat and the profitability of behavioral strategies may vary over annual cycles of ringed seal life history. For example, beginning in late spring, ringed seals undergo their annual pelage molt; an important physiological event in which several epidermal layers and the fur are shed and regenerated. This process is facilitated by infusing the epidermis with blood—providing the nutrients, oxygen, and warmth needed for tissue regeneration—but unsustainable levels of heat conduction from molting seals occurs when they are immersed in frigid Arctic waters (Boily, 1995). The high metabolic demands of the molt (Feltz & Fay, 1966; Ryg, Smith, & Øritsland, 1990) potentially set up a scenario in which ringed seals face energetic trade‐offs between foraging and hauling out. While molting, ringed seals appear to modify their behavior to compensate for their heat loss by hauling out more—particularly during the warmer midday hours in May and June (Figure 11) (Kelly et al., 2010; Kelly & Quakenbush, 1990)—and foraging less (Young & Ferguson, 2013). Behavioral strategies that lower energetic losses while simultaneously accelerating completion of the molt should be favored (Berta, Sumich, & Kovacs, 2015; McLaren, 1958), as possibly evidenced in our data by long movements to distant sea ice followed by extended haul‐out time in lieu of feeding. When considering the long‐range movements that ringed seals made to the Arctic Basin in the mid‐late summer, it seems plausible that the pursuit of available sea ice for the purpose of hauling out represents a behavioral strategy that weighs the relative quality of habitat against its value toward meeting a seal's physiological requirements.

The inclusion of the factor *CapYear*, which appeared in four of the five “best” models from our model sets (Table 3; Appendices E and F), was in response to two noteworthy events that occurred in 2011–2012. The first event was the emergence of a disease among ice seals that caused an abnormal molt, skin lesions, lethargy, mortality, and/or the unusual tendency to haul out on land (Herreman, *pers. obs.*). This disease was ultimately designated as an UME by NOAA. The second noteworthy event was the unusually early breakup of the sea ice in July of 2011, which was followed in March 2012 by the greatest sea‐ice maximum and mean sea‐ice concentrations recorded in the Bering Sea since start of the satellite record in 1979 (Fetterer et al., 2017). It is conceivable that annual variations in sea‐ice dynamics can drive physiologically mediated seal behavior. For example, given the energetic importance of hauling out during the molt, it is plausible that early sea‐ice breakup can motivate energetically depleted seals to use terrestrial haul‐outs out of necessity. Whether and to what extent the UME affected the decision for when/where to haul out cannot be ascertained given the data available. However, despite earlier sea‐ice breakup dates in both 2015 and 2017, no terrestrial haul‐out behavior was observed (A. Von Duyke, *pers. obs.*), suggesting that the UME may have affected ringed seal behavior. Though none of the seals tagged in 2011 displayed obvious symptoms of the UME at the time of capture, they were later determined to be both morphologically and behaviorally different from seals tagged in 2014 and 2016. Specifically, the 2011 seals were smaller (Table 1) and, after release, moved at higher rates, over longer durations, and ventured farther offshore (Figures 3c,d and 4; Appendix F). Ultimately, all five of the 2011 seals in this investigation made forays beyond the shelf‐break into the deep‐water Arctic Basin where they hauled out more than they foraged. The distinctive morphology, behavior, and spatial distribution of the seals tagged in 2011 do call attention to reports of two purported ringed seal ecotypes: (a) a smaller pelagic “pack‐ice seal” and (b) a larger coastal “fast‐ice seal” (Fedoseev, 1975; Finley, Miller, Davis, & Koski, 1983; Freuchen, 1935; Gorlova, Krylovich, Savinetsky, & Khasanov, 2012; McLaren, 1958). The different morphometric and behavioral characteristics of the seals tagged in 2011 were consistent with the notion of a smaller, more offshore pack‐ice ecotype. Could annual sea‐ice variability, natural life‐cycles, and energetic perturbations brought on by disease have worked together to bring a population of seals into an area they do not normally occupy, thereby making them more available for capture? If these two purported ringed seal ecotypes exist, and occupy different niches, it is plausible that one ecotype may experience and/or respond differently to ecological change. It is beyond the ability of our data to disentangle environmental variability from the possible existence and influence of ringed seal ecotypes. Nevertheless, our results are intriguing and highlight the need to better understand the population structure of ringed seals from regions that are difficult to access, as this may be important to ringed seal conservation and management, and to Arctic marine ecology.

**TABLE 3:** Top models explaining variance in movement rate, distance from the mainland, concentration of sea ice occupied, distance from sea‐ice edge, and haul‐out duration as a function of sex, age class, season, and capture year

Model set	Response variable	Model
I	MOVEMENT RATE	Season + CapYear +Season:CapYear
II	DISTANCE TO MAINLAND	CapYear
III	DISTANCE TO ICE‐EDGE	Season + CapYear
IV	ICE CONCENTRATION	AgeClass + CapYear
V	HAUL‐OUT TIME	Sex + Season +Sex:Season

Note

Underlined variables are statistically significant (*α* = 0.05). Full model sets generated by this procedure are presented in Appendix E.

Further implications of sea‐ice dynamics—particularly reductions in sea‐ice availability—may include energetic consequences due to the disruption of the relationships among access to sea ice for haul‐out, prey access, and seal physiology. Under ideal conditions, hauling out on sea ice in high‐quality foraging habitat (i.e., continental shelf) could enable molting ringed seals to partially offset energetic costs accrued from reproduction, lactation, and molting (Ryg & Øritsland, 1991), particularly if they can profitably capture prey. However, earlier northward retreat of pack‐ice (Comiso, Meier, & Gersten, 2017) may lead to overall reductions in habitat quality by shifting available sea‐ice haul‐outs to less productive off‐shelf waters (i.e., Arctic Basin). Under such conditions, ringed seals in Alaska may have to choose to: (a) forage in more productive habitat while hauling out less (Hamilton, Kovacs, Ims, & Lydersen, 2018)—potentially incurring energetic costs associated with heat loss and/or an extended molt; (b) haul out on remnants of sea ice, even if located in lower‐quality habitat—which may facilitate a faster molt, but come at the cost of fewer (i.e., lost) and/or less productive foraging opportunities; or (c) haul out on land (Lydersen, Vaquie‐Garcia, Lydersen, Christensen, & Kovacs, 2017) near higher‐quality foraging habitat—again facilitating the molt, but likely increasing predation risk. Several seals tagged in 2011 behaved in a manner consistent with the second option, though they did not venture off‐shelf until mid‐summer/early fall (Figure 5), which is well after the normal molt period. Though they exhibited some repetitive‐diving behavior (i.e., foraging), most of their time was spent hauled out on the ice. Off‐shelf forays in 2014 and 2016 were less frequent and occurred earlier in the summer (Figure 5). Currently, it is unknown whether a protracted or otherwise complicated molt (e.g., UME) could motivate seals to make late‐summer forays to the retreating pack‐ice in order to haul out. A more complete understanding of phocid molting physiology with respect to energetics may help clarify the drivers of this behavior, including the relative value of habitat over the course of a seal's annual life‐cycle. The quality of a habitat (i.e., its value to an animal's fitness) is a function of local environmental conditions and eco‐physiological constraints (Charnov, 1976; Lima, 1983), the interactions of which can shape habitat selection via the profitability of different behaviors. How this occurs may not be straightforward and, given their dynamic environment and the many possible scenarios encountered by ringed seals, is likely the net sum of numerous behavioral adjustments that optimize energy intake given the relative ratios of costs and benefits (Born et al., 2004; Ferguson & Higdon, 2006; Stephens & Krebs, 1986).

Based on their high abundance and wide distribution (Reeves, 1998), ringed seals are a very successful species, likely due to behavioral plasticity that has allowed them to exploit a variety of habitats throughout the circumpolar north. To date, ringed seals in the Bering and Chukchi seas have not exhibited declines in body condition, growth, or reproduction observed in other populations (Crawford et al., 2015). In the face of an accelerating trend toward earlier, more rapid, and/or more extensive summer sea‐ice melt (Comiso et al., 2017), as well as recent dramatic losses of winter sea ice in the Bering Sea (Siddon & Zador, 2018), a more comprehensive understanding of the energetic consequences and behavioral trade‐offs (Laidre et al., 2008) faced by ringed seals throughout their life‐cycle is needed to help guide their conservation and management.

## 5 CONCLUSIONS

This study adds to a growing body of knowledge about ringed seal movements and behaviors. Seals were captured in a region that had received little prior investigation and were instrumented with satellite transmitters capable of providing location, information about individual dives, and hourly haul‐out status. Like other ringed seal tracking studies in the Beaufort and Chukchi seas, most of the seals we tagged near Utqiaġvik moved into the southern Chukchi and Bering seas during winter. They occupied a diversity of habitats and spatial distributions, from close to shore and very localized, to far offshore and wide‐ranging in the drifting sea ice. The ringed seals we captured in 2011, concurrent with a UME that affected all ice‐seal species, were physically smaller than seals captured in other years and maintained a more pelagic distribution, raising speculation that the UME could have facilitated the tagging of a “pelagic” ringed seal ecotype that would not have otherwise been available for capture nearshore. Many ringed seals, especially those tagged in 2011, made forays into the deep Arctic Basin with an apparent intent to reach the pack‐ice to haul out. Focused bouts of repetitive diving occurred over the continental shelf for >12 hr/day, usually to depths at or near the ocean floor. Hauling out tended to be progressively more nocturnal from winter to early spring; but abruptly switched in May to a pronounced daytime haul‐out pattern with onset of the molt.

As a “threatened” species (Endangered Species Act [ESA]) (National Marine Fisheries Service, 2012), recovery criteria for ringed seals is drawn from the best available science about their habitat use and behavior; as well as knowledge about the dynamics of pinniped populations overall (Conn et al., 2014). Given the potential for increases in human/wildlife conflicts in the Arctic (Harsem et al., 2015; Smith & Stephenson, 2013), mitigation and recovery strategies for ringed seals will benefit from better information about their movements and behavior. Ongoing conservation efforts for polar bears—another ESA threatened species—will also benefit from an improved ecological understanding of ringed seals (Durner et al., 2009; Wilson, Horne, Rode, Regehr, & Durner, 2014). And, because the Arctic is a stochastic environment (Walsh, 2008) where rapid climate mediated change is already occurring (Post et al., 2013), continued research that fills gaps in poorly sampled regions will contribute to a more comprehensive understanding of the Arctic as an ecosystem, and therein the eco‐physiological processes that are important to the conservation and management of ringed seals—a vulnerable species with high ecological and cultural value (Condon, Collings, & Wenzel, 1995; Huntington, Quakenbush, & Nelson, 2016).

## CONFLICT OF INTEREST

The authors declare no conflict of interest.

## AUTHOR CONTRIBUTION

**Andrew L. Von Duyke:** Conceptualization (lead); Data curation (equal); Formal analysis (equal); Funding acquisition (lead); Investigation (lead); Methodology (equal); Project administration (lead); Resources (lead); Software (equal); Supervision (lead); Validation (equal); Visualization (equal); Writing‐original draft (lead); Writing‐review & editing (lead). **David C. Douglas:** Conceptualization (equal); Data curation (equal); Formal analysis (equal); Funding acquisition (supporting); Investigation (supporting); Methodology (equal); Project administration (supporting); Resources (supporting); Software (equal); Supervision (supporting); Validation (equal); Visualization (equal); Writing‐original draft (supporting); Writing‐review & editing (supporting). **Jason K. Herreman:** Conceptualization (supporting); Data curation (supporting); Formal analysis (supporting); Funding acquisition (equal); Investigation (equal); Methodology (supporting); Project administration (equal); Resources (supporting); Software (supporting); Supervision (equal); Validation (supporting); Visualization (supporting); Writing‐original draft (supporting); Writing‐review & editing (supporting). **Justin A. Crawford:** Conceptualization (equal); Data curation (equal); Formal analysis (supporting); Funding acquisition (supporting); Investigation (equal); Methodology (equal); Project administration (supporting); Resources (equal); Software (equal); Supervision (supporting); Validation (supporting); Visualization (supporting); Writing‐original draft (supporting); Writing‐review & editing (supporting). 

## Supporting information

Appendix A‐G

## Data Availability

The data set analyzed for this study is available from the Dryad Digital Repository https://doi.org/10.5061/dryad.zpc866t65 (Von Duyke, Douglas, Herreman, & Crawford, 2020).
