# Supplementary material for: Ringed seal (Pusa hispida) seasonal movements, diving, and haul‐out behavior in the Beaufort, Chukchi, and Bering Seas (2011–2017)
Source: Ecol Evol. 2020 May 5;10(12):5595–616. doi: 10.1002/ece3.6302 (PMC7319173; doi:10.1002/ece3.6302)
Supplement: Supplementary file 1 — Appendix A‐G [file ECE3-10-5595-s001.docx]

**APPENDICES**

**APPENDIX A** Ringed seals captured and instrumented with Argos satellite transmitters near Utqiaġvik, Alaska, during 2011–2016 by the North Slope Borough Department of Wildlife Management (*n* = 39). The 17 seals presented in this paper are denoted with gray shading. Last location dates are based on filtered data. Tags deployed in 2013 were believed to have firmware problems and were omitted from analyses in this paper. Splash and SPOT tag models were manufactured by Wildlife Computers (Redmond WA, USA) and CTD tags by Sea Mammal Research Unit (St. Andrews, Scotland).

| **seal ID** | **sex** | **weight**  **(kg)** | **length**  **(cm)** | **ax. girth**  **(cm)** | **claw**  **bands** | **age** | **tag_ID** | **tag**  **model** | **capture**  **date** | **last**  **loc** | **elapsed**  **days** |
| --- | --- | --- | --- | --- | --- | --- | --- | --- | --- | --- | --- |
| PH2011BW01 | M | 16.5 | 87 | 68 | 2+ | Subadult | 106411 | SPOT | 13-Jul-2011 | 13-Jul-2011 | 0 |
| PH2011BW02 | M | 30.4 | 95 | 85 | 5+ | Adult | 106427 | Splash | 14-Jul-2011 | 20-Jul-2011 | 6 |
|  |  |  |  |  |  |  | 106409 | SPOT |  | 13-Nov-2013 | 853^a^ |
| PH2011BW03 | M | 24.8 | 103 | 84 | 4+ | Subadult | 106429 | Splash | 15-Jul-2011 | 10-Jun-2012 | 331 |
|  |  |  |  |  |  |  | 106408 | SPOT |  | 29-May-2012 | 319 |
| PH2011BW04 | M | 30.1 | 100 | 79 | 4+ | Subadult | 106434 | Splash | 15-Jul-2011 | 30-Jul-2011 | 15 |
|  |  |  |  |  |  |  | 106413 | SPOT |  | 18-Sep-2011 | 65 |
| PH2011BW05 | M | 14.5 | 68 | 71 | 1+ | Pup/Sub | 106424 | Splash | 16-Jul-2011 | 07-Sep-2011 | 53 |
| PH2011BW06 | M | 16 | 88 | 66 | 1+ | Subadult | 106423 | Splash | 17-Jul-2011 | 19-Jul-2011 | 2 |
|  |  |  |  |  |  |  | 106415 | SPOT |  |  | 0 |
| PH2011BW07 | F | 11.6 | 69 | 62 | 0+ | Pup | 106435 | Splash | 18-Jul-2011 | 14-Aug-2011 | 27 |
| PH2011BW08 | F | 21.7 | 90 | 74 | 8+ | Adult | 106417 | SPOT | 19-Jul-2011 | 20-Jul-2011 | 1 |
| PH2011BW09 | M | 11.9 | 67 | 58 | 0+ | Pup | 106430 | Splash | 20-Jul-2011 | 25-Jul-2011 | 5 |
| PH2011BW10 | F | 26.6 | 92 | 72 | 8+ | Adult | 106420 | Splash | 20-Jul-2011 | 02-May-2012 | 287 |
|  |  |  |  |  |  |  | 106416 | SPOT |  | 17-Aug-2011 | 28 |
| PH2011BW11 | F | 23.2 | 93 | 76 | 7+ | Adult | 106425 | Splash | 21-Jul-2011 | 05-Jun-2012 | 320 |
|  |  |  |  |  |  |  | 106414 | SPOT |  | 05-Nov-2011 | 107 |
| PH2011BW12 | M | 27.2 | 92 | 81 | 8+ | Adult | 106428 | Splash | 21-Jul-2011 | 05-May-2012 | 289 |
|  |  |  |  |  |  |  | 106407 | SPOT |  | 05-Nov-2011 | 107 |
| PH2011BW13 | M | 34.8 | 91 | 84 | 8+ | Adult | 106422 | Splash | 21-Jul-2011 | 12-Jan-2012 | 175 |
|  |  |  |  |  |  |  | 106410 | SPOT |  | 05-Aug-2011 | 15 |
| PH2011BW14 | F | 21.3 | 92 | 75 | 7+ | Adult | 106431 | Splash | 22-Jul-2011 | 30-Jul-2011 | 8 |
|  |  |  |  |  |  |  | 106405 | SPOT |  | 24-Jul-2011 | 2 |
| PH2011BW15 | M | 34.2 | 116 | 85 | 9+ | Adult | 106412 | SPOT | 29-Jul-2011 | 05-Aug-2011 | 7 |
| PH2011BW16 | F | 8.7 | 67 | 56 | 0+ | Pup | 106432 | Splash | 08-Aug-2011 | 09-Aug-2011 | 1 |
| PH2011BW17 | F | 17 | 81 | 70 | 1+ | Subadult | 106419 | SPOT | 09-Aug-2011 | 12-Oct-2011 | 64 |
| PH2011BW18 | M | 29.8 | 109 | 81 | 8+ | Adult | 106404 | SPOT | 10-Aug-2011 | 11-Aug-2011 | 1 |
| PH2011BW19 | M | 27.9 | 94 | 83 | 8+ | Adult | 106406 | SPOT | 11-Aug-2011 | 14-Sep-2011 | 34 |
| PH2011BW20 | F | 23.8 | 91 | 69 | 9+ | Adult | 106418 | SPOT | 12-Aug-2011 | 26-Sep-2011 | 45 |
| PH2011BW21 | F | 12.9 | 77 | 60 | 0+ | Pup | 106421 | Splash | 29-Sep-2011 | 01-Oct-2011 | 2 |
| PH2013BW01 | F | 35.7 |  |  |  | Adult | 118087 | Splash | 17-Jul-2013 | 24-Aug-2013 | 38 |
|  |  |  |  |  |  |  | 118115 | SPOT |  | 20-Jan-2014 | 187 |
| PH2013BW02 | F | 35.7 |  |  | 6+ | Adult | 118091 | Splash | 19-Jul-2013 | 22-Jul-2013 | 3 |
| PH2013BW03 | F | 19.2 |  |  | 0+ | Pup | 118088 | Splash | 04-Sep-2013 | 23-Oct-2013 | 49 |
|  |  |  |  |  |  |  | 118121 | SPOT |  |  | 0 |
| PH2014BW01 | M | 53.6 | 100 | 95 | 6+ | Adult | 118094 | Splash | 21-Jul-2014 | 14-May-2015 | 297 |
|  |  |  |  |  |  |  | 118124 | SPOT |  | 20-May-2015 | 303 |
| PH2014BW02 | M | 18.3 | 74 | 70 |  | Subadult | 118098 | Splash | 22-Jul-2014 | 03-Feb-2015 | 196 |
|  |  |  |  |  |  |  | 118116 | SPOT |  | 30-Nov-2014 | 131 |
| PH2014BW03 | F | 9.4 | 68 | 55 | 0 | Pup | 118100 | Splash | 30-Aug-2014 | 03-Sep-2014 | 4 |
| PH2016BW01 | M | 50.9 | 110 | 101 | 6+ | Adult | 149357 | Splash | 01-Jul-2016 | 11-Feb-2017 | 225 |
|  |  |  |  |  |  |  | 149400 | SPOT |  | 30-Oct-2017 | 486 |
| PH2016BW03 | F | 24.8 | 86 | 81 | 1+ | Subadult | 149371 | Splash | 01-Jul-2016 | 27-Jan-2017 | 210 |
|  |  |  |  |  |  |  | 149390 | SPOT |  | 24-Jan-2017 | 207 |
| PH2016BW04 | M | 49.1 | 114 | 101 | 6+ | Adult | 149451 | Splash | 01-Jul-2016 | 23-Mar-2017 | 265 |
|  |  |  |  |  |  |  | 149394 | SPOT |  | 23-Mar-2017 | 265 |
| PH2016BW05 | F | 41.3 | 104 | 102 | 5+ | Adult | 149372 | Splash | 01-Jul-2016 | 05-Jul-2016 | 4 |
|  |  |  |  |  |  |  | 149382 | SPOT |  | 07-Aug-2016 | 37 |
| PH2016BW06 | F | 25.9 | 86 | 84 | 1 | Subadult | 149366 | Splash | 01-Jul-2016 | 22-Jan-2017 | 205 |
|  |  |  |  |  |  |  | 149383 | SPOT |  | 07-Sep-2016 | 68 |
| PH2016BW08 | F | 46.0 | 90 | 100 | 6+ | Adult | 149370 | Splash | 02-Jul-2016 | 19-Jul-2016 | 17 |
|  |  |  |  |  |  |  | 149384 | SPOT |  | 12-Nov-2016 | 133 |
| PH2016BW09 | F | 46.7 | 113 | 101 | 5+ | Adult | 149363 | Splash | 02-Jul-2016 | 24-Feb-2017 | 237 |
|  |  |  |  |  |  |  | 149393 | SPOT |  | 29-Aug-2016 | 58 |
| PH2016BW10 | F | 40.0 | 100 | 92 | 4 | Subadult | 149355 | Splash | 02-Jul-2016 | 01-Feb-2017 | 223 |
|  |  |  |  |  |  |  | 149385 | SPOT |  | 15-Aug-2016 | 44 |
| PH2016BW11 | M | 36.6 | 98 | 92 | 5+ | Adult | 149361 | Splash | 02-Jul-2016 | 26-Feb-2017 | 239 |
|  |  |  |  |  |  |  | 149395 | SPOT |  | 16-Dec-2016 | 167 |
| PH2016BW12 | M | 36.8 | 103 | 93 | 6+ | Adult | 149365 | Splash | 02-Jul-2016 | 07-Jan-2017 | 189 |
|  |  |  |  |  |  |  | 149381 | SPOT |  | 06-Jan-2017 | 188 |
| PH16BRW-120350 | M | 51.6 | 112 | 124 | 8+ | Adult | 120350 | CTD | 01-Jul-2016 | 07-Apr-2017 | 280 |
|  |  |  |  |  |  |  | 149388 | SPOT |  | 11-Jan-2017 | 194 |
| PH16BRW-120353 | M | 51.6 | 112 | 105 | 7+ | Adult | 120353 | CTD | 01-Jul-2016 | 20-Oct-2016 | 111 |
|  |  |  |  |  |  |  | 149398 | SPOT |  | 01-Feb-2017 | 215 |

^a^ includes a 513-day data gap from June 2012 to November 2013

**APPENDIX B** Method of filtering implausible Argos locations

Implausible Argos locations were excluded using the Douglas Argos Filter (Douglas et al. 2012), which judges location quality, movement rates, distances, and turning angles. High quality locations (Argos classes 1, 2, or 3) were retained unconditionally. Low quality locations (Argos classes 0, A, B, and Z) within 5 km of a preceding or subsequent retained location were also retained owing to spatial redundancy. Any remaining low quality locations were included only if the resultant movement rates were <5.6 m·s^-1^ and the internal angles (α, in degrees) formed by preceding and subsequent vectors (of lengths d_1_ and d_2_ km) were not suspiciously acute (α >-25 + β × ln[minimum (d_1_,d_2_)], where β = 25). We assigned β = 25 because it performed well for our specific tracking data across seasons and regions.

Because our filtering objective was to prepare tracking data for analysis with the continuous-time correlated random walk model (CRAWL), we applied a less restrictive movement rate threshold (5.6 m·s^-1^ = 20 km·h^-1^) than has been commonly applied (~2.5 m·s^-1^) for direct analyses of Argos seal tracking data (e.g., Lowry et al. 1998, Crawford et al. 2012). Our strategy was to exclude the more inaccurate locations (Johnson et al. 2008), while tempering any undue inflation of temporal gaps in the tracking time series (Lonergan, Fedak, & McConnell 2009). Among the CRAWL-estimated locations analyzed in this paper (i.e., estimates every 6 hours), the median movement rate was 0.7 km·h^-1^, with an interquartile range of 0.3–1.5, a 99^th^ percentile of 4.5 km·h^-1^, and a maximum of 10.5 km·h^-1^.

**APPENDIX C** Graphical illustration of dive patterns associated with three dive-behavior classes

Dive behavior classifications are illustrated in a subset of the dive-behavior time-series data for one ringed seal during an 18-hour period spanning December 29 – 30, 2016 (UTC). Color bars across the top of the figure indicate the three behavioral classes: repetitive-diving to similar depths (red), mixed-diving to dissimilar depths (blue), and resting at the surface for a duration ≥10 minutes (yellow). The locations for this seal occurred in a region where the ocean was 35 – 40 m deep, suggesting that most repetitive-dives were bottom-dives. Gaps in the dive-behavior time series were due to bandwidth constraints of the Argos System, intermittent satellite availability, and pre-defined transmission limits imposed on tags to conserve battery life.


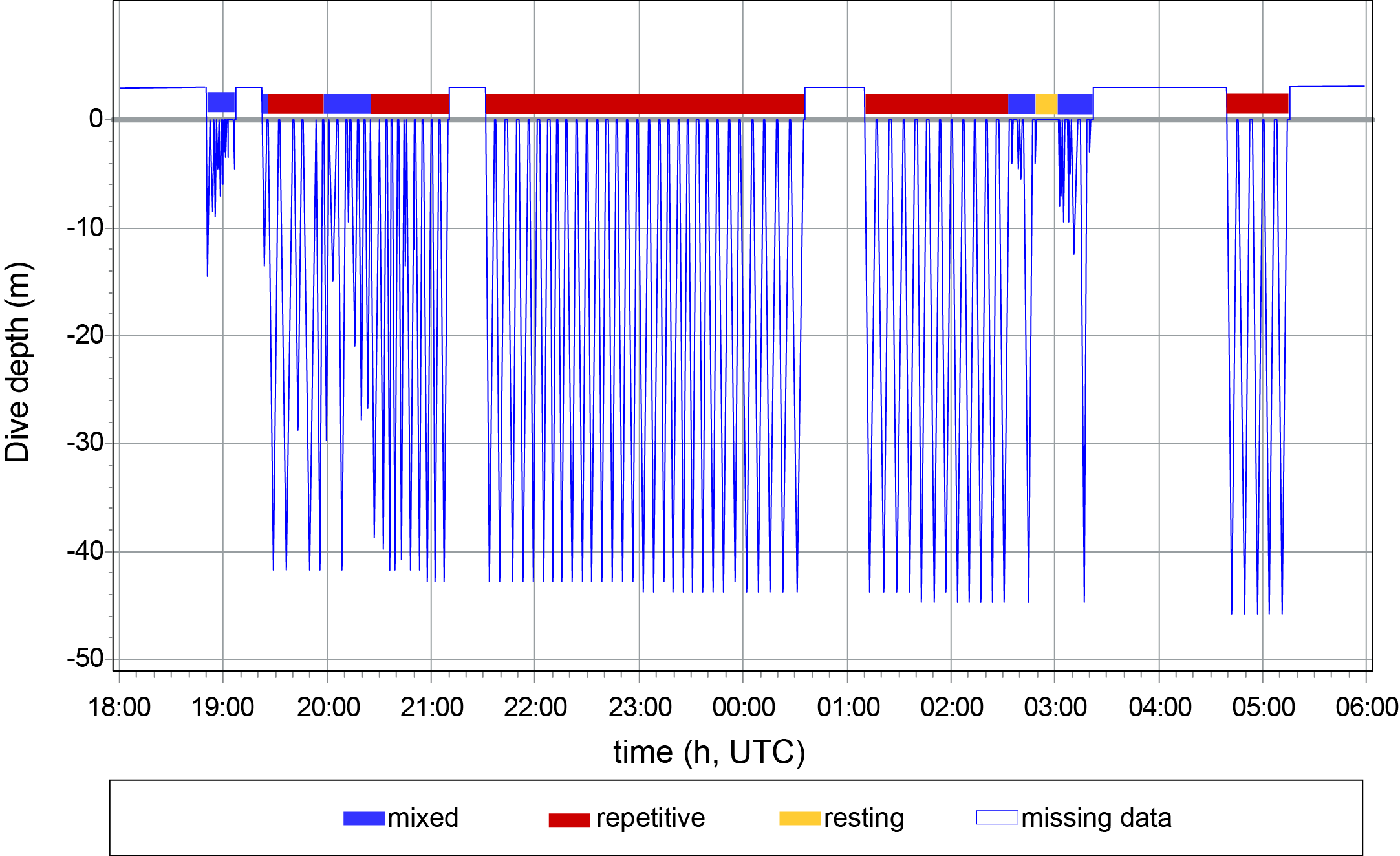


**APPENDIX D** Validating unbiased dive behavior

Unbiased estimates of daily activity budgets assume that the dive-behavior time series reported sampling uniformly across all hours of the day. To test this assumption, we summarized the percentage of dive-behavior data collected during each hour of the day obtained from 8 SPLASH tags deployed in 2016. The blue points show the mean percentage of acquired dive-behavior data (±1 SE) for each hour of the day, and the gray lines show the values for each individual tag. We conclude that the dive-behavior data acquired from these 8 SPLASH tags achieved a reasonably uniform sampling distribution of ~25% across all hours of the day. This uniform distribution was achieved, in part, by provisioning the SPLASH tag’s “Transmission Control” option to transmit data collected over the last 4 days. By transmitting data drawn from a 4-day pool, we were able to overcome short-term (i.e, daily scale) temporal biases in relaying data through the Argos System that arise from diel patterns in satellite coverage.


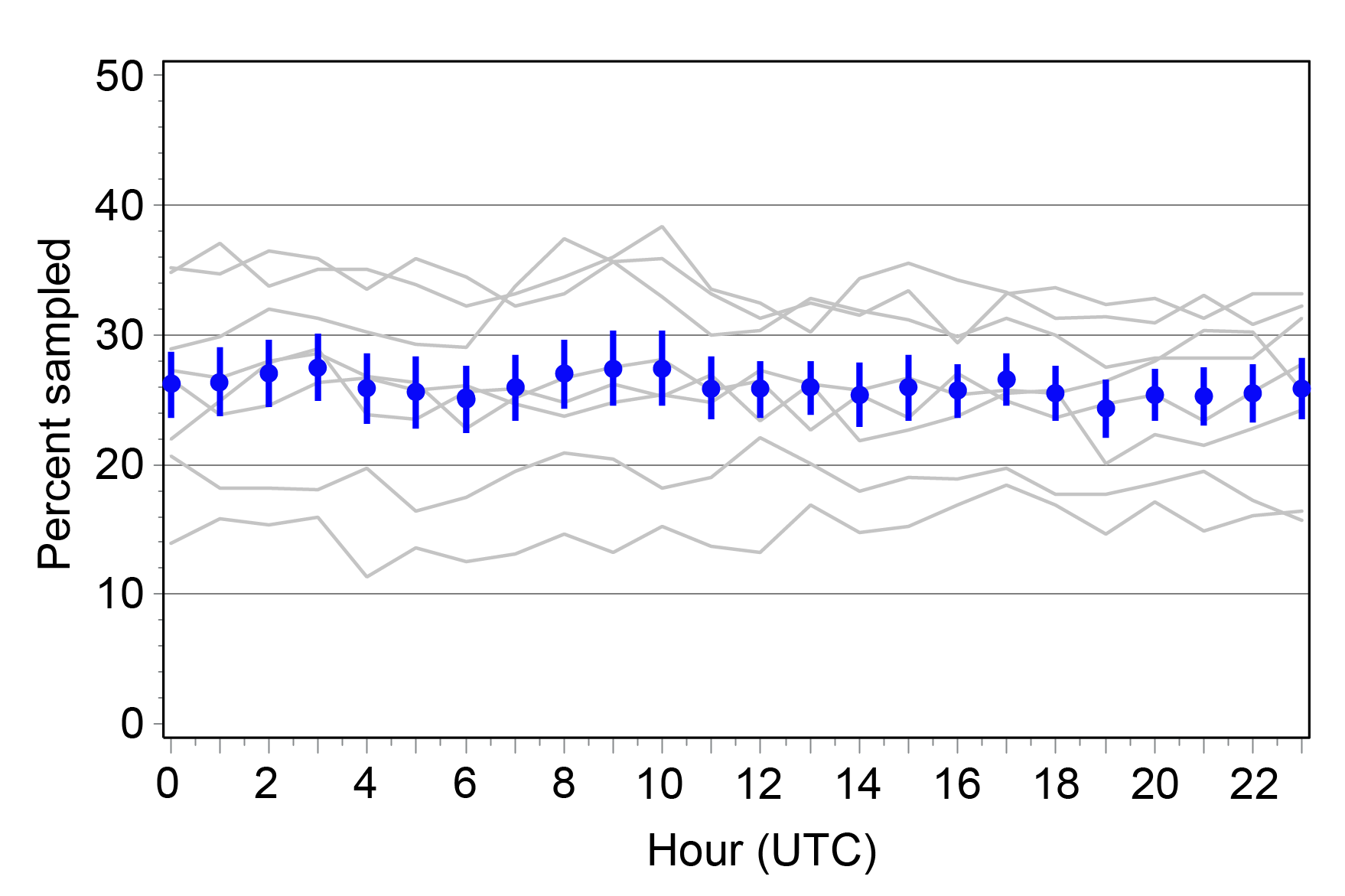


**APPENDIX E** Full model sets

AIC_C_ rankings for the full set of models in each of five model sets designed to examine variation in (I) movement rates, (II) distance from the mainland, (III) distance to sea-ice edge, (IV) concentration of sea-ice occupied, and (V) haul-out duration as a function of *Sex* (FEMALE, MALE), *AgeClass* (ADULT, SUBADULT), *Season* (ICE-COVERED, OPEN-WATER), and *CapYear* (seals captured in 2011 versus those captured in 2014 and 2016). The most parsimonious models, shown in bold font, are also reported in Table 3. Underlined variables indicate that the coefficient was statistically significant (p ≤ 0.05). Gray shading indicates ΔAIC_C_ ≤ 2.

| **I.** | **MOVEMENT RATE** | **AIC_C_** | **ΔAIC_C_** | ***w*** |
| --- | --- | --- | --- | --- |
| **I.01** | **Season + CapYear + Season:CapYear** | **16211.12** | **0.00** | **0.65** |
| I.02 | Season + CapYear + AgeClass + Season:CapYear | 16212.35 | 1.23 | 0.35 |
| I.03 | Season + CapYear | 16266.67 | 55.56 | 0.00 |
| I.04 | Season + CapYear + AgeClass + Season:AgeClass | 16267.00 | 55.88 | 0.00 |
| I.05 | Season + CapYear + AgeClass | 16267.68 | 56.56 | 0.00 |
| I.06 | Season | 16268.34 | 57.22 | 0.00 |
| I.07 | Season + CapYear + Sex | 16268.68 | 57.56 | 0.00 |
| I.08 | Season + CapYear + AgeClass + AgeClass:CapYear | 16269.31 | 58.20 | 0.00 |
| I.09 | Season + CapYear + Sex + AgeClass | 16269.54 | 58.42 | 0.00 |
| I.10 | Season + AgeClass | 16269.93 | 58.81 | 0.00 |
| I.11 | Season + Sex | 16270.33 | 59.21 | 0.00 |
| I.12 | Season + Sex + AgeClass | 16271.92 | 60.81 | 0.00 |
| I.13 | CapYear | 16994.72 | 783.60 | 0.00 |
| I.14 | CapYear + AgeClass | 16995.08 | 783.96 | 0.00 |
| I.15 | AgeClass | 16995.19 | 784.07 | 0.00 |
| I.16 | Sex | 16996.13 | 785.01 | 0.00 |
| I.17 | Sex + CapYear | 16996.63 | 785.51 | 0.00 |
| I.18 | Sex + AgeClass + CapYear | 16997.07 | 785.95 | 0.00 |
| I.19 | Sex + AgeClass | 16997.19 | 786.07 | 0.00 |

| **II.** | **DISTANCE TO MAINLAND** | **AIC_C_** | **ΔAIC_C_** | ***w*** |
| --- | --- | --- | --- | --- |
| **II.01** | **CapYear** | **11507.14** | **0.00** | **0.28** |
| II.02 | CapYear + Season | 11508.58 | 1.44 | 0.14 |
| II.03 | CapYear + Sex | 11508.86 | 1.73 | 0.12 |
| II.04 | CapYear + AgeClass | 11508.96 | 1.83 | 0.11 |
| II.05 | CapYear + Season + Sex | 11510.31 | 3.17 | 0.06 |
| II.06 | CapYear + Season + AgeClass | 11510.41 | 3.28 | 0.06 |
| II.07 | CapYear + Sex + AgeClass | 11510.51 | 3.38 | 0.05 |
| II.08 | CapYear + Season + CapYear:Season | 11510.52 | 3.39 | 0.05 |
| II.09 | CapYear + Sex + CapYear:Sex | 11510.84 | 3.70 | 0.04 |
| II.10 | CapYear + AgeClass + CapYear:AgeClass | 11510.97 | 3.83 | 0.04 |
| II.11 | CapYear + AgeClass + Sex + Season | 11511.96 | 4.83 | 0.03 |
| II.12 | Season | 11516.05 | 8.92 | 0.00 |
| II.13 | AgeClass | 11516.54 | 9.41 | 0.00 |
| II.14 | Sex | 11516.58 | 9.44 | 0.00 |
| II.15 | AgeClass + Season | 11518.02 | 10.88 | 0.00 |
| II.16 | Sex + Season | 11518.05 | 10.92 | 0.00 |
| II.17 | Sex + AgeClass | 11518.53 | 11.40 | 0.00 |
| II.18 | Sex + AgeClass + Season | 11520.01 | 12.87 | 0.00 |

| **III.** | **DISTANCE TO ICE EDGE** | **AIC_C_** | **ΔAIC_C_** | ***w*** |
| --- | --- | --- | --- | --- |
| **III.01** | **Season + CapYear** | **14360.90** | **0.00** | **0.22** |
| III.02 | Season + CapYear + AgeClass | 14362.28 | 1.38 | 0.11 |
| III.03 | Season + CapYear + Season:CapYear | 14362.91 | 2.00 | 0.08 |
| III.04 | Season + CapYear + Sex | 14362.91 | 2.00 | 0.08 |
| III.05 | CapYear | 14362.97 | 2.06 | 0.08 |
| III.06 | Season | 14363.21 | 2.31 | 0.07 |
| III.07 | Season + CapYear + AgeClass + Season:AgeClass | 14363.95 | 3.04 | 0.05 |
| III.08 | Season + CapYear + AgeClass + Sex | 14364.21 | 3.31 | 0.04 |
| III.09 | Season + CapYear + AgeClass + CapYear:AgeClass | 14364.24 | 3.33 | 0.04 |
| III.10 | Season + CapYear + AgeClass + Season:CapYear | 14364.29 | 3.38 | 0.04 |
| III.11 | CapYear + AgeClass | 14364.41 | 3.51 | 0.04 |
| III.12 | Season + AgeClass | 14364.50 | 3.59 | 0.04 |
| III.13 | CapYear + Sex | 14364.97 | 4.07 | 0.03 |
| III.14 | Season + Sex | 14365.11 | 4.21 | 0.03 |
| III.15 | AgeClass | 14366.13 | 5.23 | 0.02 |
| III.16 | Season + Sex + AgeClass | 14366.19 | 5.29 | 0.02 |
| III.17 | CapYear + Sex + AgeClass | 14366.36 | 5.45 | 0.01 |
| III.18 | Sex | 14366.70 | 5.80 | 0.01 |
| III.19 | Sex + AgeClass | 14367.87 | 6.97 | 0.01 |

| **IV.** | **ICE CONCENTRATION** | **AIC_C_** | **ΔAIC_C_** | ***W*** |
| --- | --- | --- | --- | --- |
| **IV.01** | **CapYear + AgeClass** | **-1405.14** | **0.00** | **0.21** |
| IV.02 | AgeClass | -1404.01 | 1.05 | 0.12 |
| IV.03 | CapYear + AgeClass + Sex | -1403.99 | 1.15 | 0.12 |
| IV.04 | CapYear | -1403.94 | 1.20 | 0.12 |
| IV.05 | CapYear + Sex | -1403.48 | 1.66 | 0.09 |
| IV.06 | CapYear + AgeClass + CapYear:AgeClass | -1403.40 | 1.75 | 0.09 |
| IV.07 | CapYear + Sex + CapYear:Sex | -1402.80 | 2.33 | 0.07 |
| IV.08 | AgeClass + Sex | -1402.41 | 2.74 | 0.05 |
| IV.09 | CapYear + AgeClass + Sex + CapYear:Sex | -1402.24 | 2.90 | 0.05 |
| IV.10 | CapYear + AgeClass + Sex + CapYear:AgeClass | -1401.99 | 3.16 | 0.04 |
| IV.11 | Sex | -1401.81 | 3.34 | 0.04 |

| **V.** | **HAUL-OUT TIME** | **AIC_C_** | **ΔAIC_C_** | ***w*** |
| --- | --- | --- | --- | --- |
| **V.01** | **Sex + Season + Sex:Season** | **6374.11** | **0.00** | **0.74** |
| V.02 | Sex + AgeClass + Sex:AgeClass | 6378.67 | 4.55 | 0.08 |
| V.03 | Sex | 6380.51 | 6.40 | 0.03 |
| V.04 | Sex + Season | 6381.15 | 7.04 | 0.02 |
| V.05 | Season | 6381.21 | 7.09 | 0.02 |
| V.06 | Sex + CapYear + Sex:CapYear | 6381.63 | 7.52 | 0.02 |
| V.07 | Sex + AgeClass | 6381.96 | 7.84 | 0.01 |
| V.08 | Sex + CapYear | 6382.48 | 8.36 | 0.01 |
| V.09 | Sex + Season + AgeClass | 6382.53 | 8.42 | 0.01 |
| V.10 | AgeClass | 6382.61 | 8.50 | 0.01 |
| V.11 | CapYear | 6382.70 | 8.59 | 0.01 |
| V.12 | Sex + CapYear + Season | 6383.07 | 8.95 | 0.01 |
| V.13 | Season + AgeClass | 6383.07 | 8.96 | 0.01 |
| V.14 | Season + CapYear | 6383.16 | 9.05 | 0.01 |
| V.15 | Sex + CapYear + AgeClass | 6384.00 | 9.90 | 0.01 |
| V.16 | Sex + Season + CapYear + AgeClass | 6384.54 | 10.43 | 0.00 |
| V.17 | CapYear + AgeClass | 6384.63 | 10.51 | 0.00 |
| V.18 | Season + CapYear + AgeClass | 6385.07 | 10.95 | 0.00 |

**APPENDIX F** Pairwise contrasts and estimated marginal means for model sets I-V

The best models from each model set in Appendix E were used to estimate the marginal mean values (right) and to produce statistical pairwise comparisons (left). These results are graphically depicted in Figure 4. P-values are adjusted according to the Tukey method, and all estimated marginal means were back-transformed prior to reporting. The factor for *Sex* is designated by FEMALE and MALE, *AgeClass* by ADULT and SUBADULT, *Season* by ICE and OPEN, and *CapYear* by 2011 and 2014.16. Note that model sets I and V included an interaction, as designated by a tilde (~). Model sets II, III, and IV include only main effects, which are separated by a dashed line when more than one. Note that negative distances to the ice edge (model set III) indicate locations inside the pack-ice, whereas positive distances indicate locations outside the pack-ice.

|  | | | | **Estimated Marginal Means** | | |
| --- | --- | --- | --- | --- | --- | --- |
| 1. **MOVEMENT RATE (km/day)** | | | | **mean** | **CI _lower_** | **CI _upper_** |
| **ICE_2011_** | *p* = 0.011* | *p* < 0.001* | *p* = 0.071 | 14.52 | 10.37 | 19.36 |
|  | **ICE_2014.16_** | *p* < 0.001* | *p* < 0.001* | 6.76 | 4.88 | 9.00 |
| ***Season ~ CapYear*** | | **OPEN_2011_** | *p* = 0.901 | 24.30 | 18.84 | 30.47 |
|  |  |  | **OPEN_2014.16_** | 22.09 | 18.66 | 25.91 |
| 1. **DISTANCE TO MAINLAND (km)** | | | | **mean** | **CI _lower_** | **CI _upper_** |
| ***CapYear*** | | **2011** | *p* = 0.005* | 120.56 | 77.26 | 173.45 |
|  |  | **2014.16** |  | 46.51 | 28.09 | 69.56 |
| 1. **DISTANCE TO ICE EDGE (km)** | | | | **mean** | **CI _lower_** | **CI _upper_** |
| ***Season*** | | **ICE** | *p* = 0.029* | 27.61 | -21.11 | 80.25 |
|  |  | **OPEN** |  | 57.21 | 6.25 | 112.09 |
| ***CapYear*** | | **2011** | *p* = 0.048* | -4.16 | -75.00 | 76.36 |
|  |  | **2014.16** |  | 96.01 | 34.89 | 162.25 |
| 1. **ICE CONCENTRATION (%)** | | | | **mean** | **CI _lower_** | **CI _upper_** |
| ***AgeClass*** | | **ADULT** | *p* = 0.060 | 73.98 | 65.41 | 81.05 |
|  |  | **SUBADULT** |  | 57.32 | 40.06 | 72.95 |
| ***CapYear*** | | **2011** | *p* = 0.067 | 73.30 | 59.05 | 83.94 |
|  |  | **2014.16** |  | 58.18 | 47.13 | 68.44 |
| 1. **HAUL-OUT TIME (hrs/haul-out)** | | | | **mean** | **CI _lower_** | **CI _upper_** |
| **FEMALE_ICE_** | *p* = 0.377 | *p* = 0.087 | *p* = 0.505 | 7.24 | 6.17 | 8.50 |
|  | **FEMALE_OPEN_** | *p* = 0.318 | *p* = 0.889 | 6.69 | 5.75 | 7.85 |
| ***Sex ~ Season*** | | **MALE_ICE_** | *p* = 0.026* | 5.64 | 4.90 | 6.42 |
|  |  |  | **MALE_OPEN_** | 6.23 | 5.47 | 7.10 |

**APPENDIX G** Forays into the Arctic Basin

Most forays into the deep-water Arctic Basin included days when seals hauled out on sea-ice (see Fig. 5), as deduced from the hourly summaries of sensor data indicating that the tracking tag was dry for >80% of a respective hour. Mean hours per day hauled out are shown for just those days during a foray that the seal hauled out for ≥1 hour. Letter in first column references the corresponding map in Fig. 5.

|  |  | **Foray into ocean >1000 m deep** | | | **# days w/** | **# days w/ >1** | **hours/day hauled-out** | | | | |
| --- | --- | --- | --- | --- | --- | --- | --- | --- | --- | --- | --- |
| **Map** | **ringed seal** | **start** | **end** | **# days** | **sensor data** | **haul-out hour** | ***x̄*** | **sd** | **min** | **max** | **n** |
| a | PH2011BW13 | 8/2/2011 | 8/11/2011 | 10 | 10 | 5 | 9.6 | 4.7 | 3 | 15 | 5 |
| b | PH2011BW10 | 8/13/2011 | 8/21/2011 | 9 | 8 | 2 | 16 | 9.9 | 9 | 23 | 2 |
| c | PH2011BW13 | 8/12/2011 | 8/22/2011 | 11 | 11 | 2 | 14 | 2.8 | 12 | 16 | 2 |
| d | PH2011BW12 | 8/16/2011 | 8/22/2011 | 7 | 6 | 2 | 15 | 4.2 | 12 | 18 | 2 |
| e | PH2011BW11 | 8/22/2011 | 8/28/2011 | 7 | 7 | 3 | 10 | 6.2 | 3 | 15 | 3 |
| e | PH2011BW03 | 8/22/2011 | 8/27/2011 | 6 | 6 | 3 | 14.7 | 6.1 | 8 | 20 | 3 |
| f | PH2011BW03 | 9/18/2011 | 9/27/2011 | 10 | 9 | 0 | -- | -- | -- | -- | -- |
| g | PH2011BW12 | 10/5/2011 | 10/15/2011 | 11 | 10 | 5 | 18.4 | 4.7 | 11 | 24 | 5 |
| h | PH2011BW13 | 10/7/2011 | 10/14/2011 | 8 | 8 | 0 | -- | -- | -- | -- | -- |
| i | PH2014BW02 | 8/8/2014 | 8/11/2014 | 4 | 3 | 2 | 9.5 | 3.5 | 7 | 12 | 2 |
| j | PH2016BW06 | 7/4/2016 | 7/18/2016 | 15 | 13 | 13 | 10.4 | 6.5 | 1 | 21 | 13 |
| j | PH2016BW09 | 7/9/2016 | 7/13/2016 | 5 | 5 | 5 | 15.8 | 6.9 | 7 | 23 | 5 |
| j | PH2016BW10 | 7/9/2016 | 7/18/2016 | 10 | 8 | 6 | 14 | 6.2 | 7 | 22 | 6 |
| k | PH2016BW01 | 8/8/2016 | 8/9/2016 | 2 | 2 | 0 | -- | -- | -- | -- | -- |
| l | PH2016BW12 | 8/9/2016 | 8/14/2016 | 6 | 5 | 3 | 11.3 | 6.1 | 6 | 18 | 3 |
| na | PH2014BW01 | 8/14/2014 | 8/17/2014 | 4 | no data | -- | -- | -- | -- | -- | -- |
| na | PH2016BW03 | 8/6/2016 | 8/7/2016 | 2 | 2 | 1 | 1 | -- | 1 | 1 | 1 |
