# Supplementary figures and images for: Ringed seal (Pusa hispida) seasonal movements, diving, and haul‐out behavior in the Beaufort, Chukchi, and Bering Seas (2011–2017)
Source: Ecol Evol. 2020 May 5;10(12):5595–616. doi: 10.1002/ece3.6302 (PMC7319173; doi:10.1002/ece3.6302)

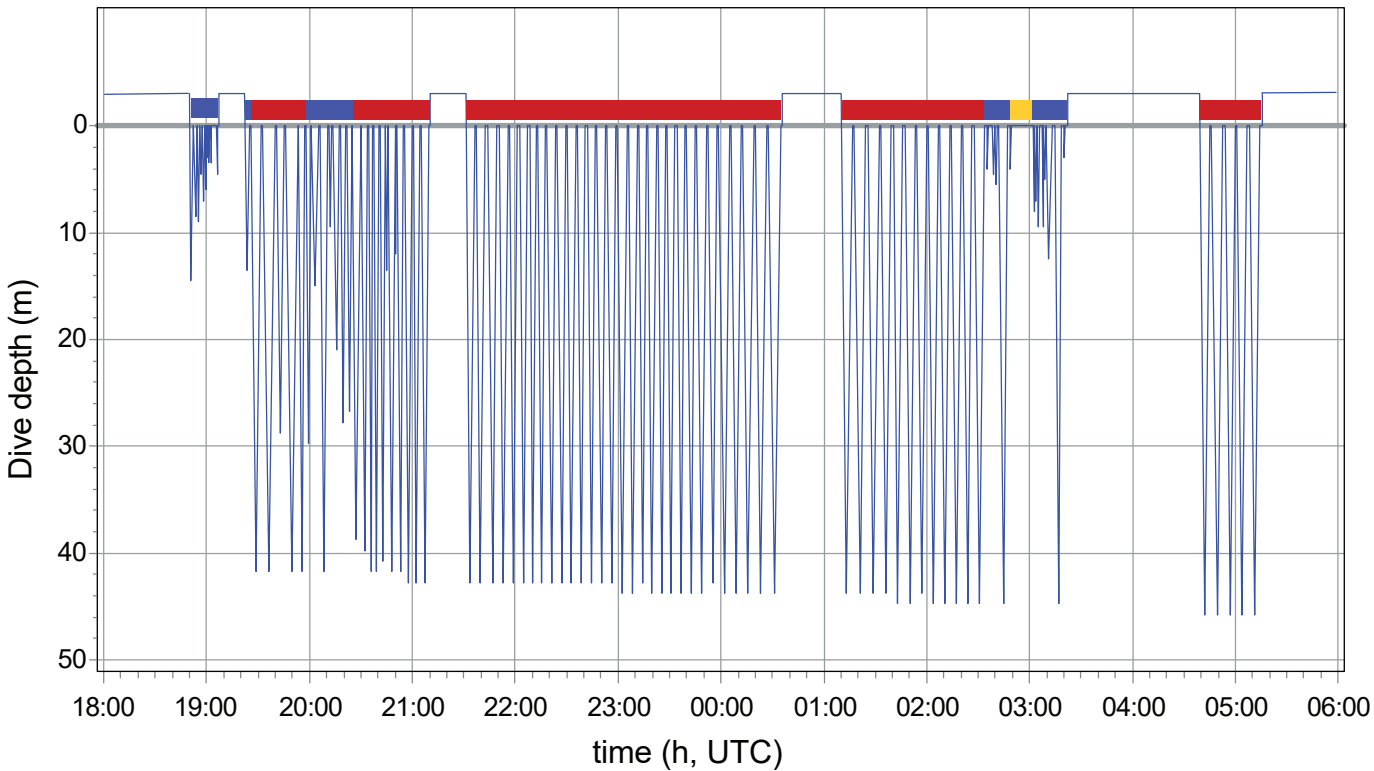

mixed

repetitive

resting

missing data

Supplement: Supplementary file 2 [file ECE3-10-5595-s002.pdf]

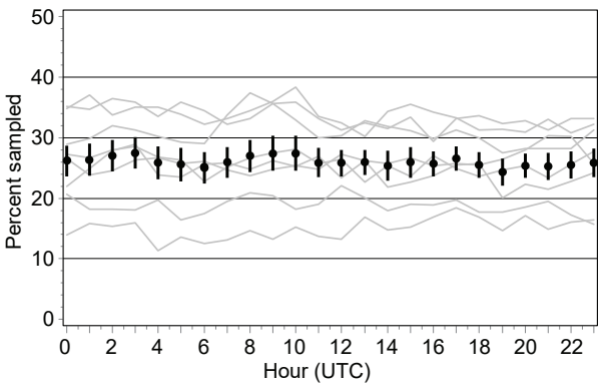

Supplement: Supplementary file 3 [file ECE3-10-5595-s003.pdf]
